# The Role of Therapeutic Endoscopic Ultrasound in Management of Malignant Double Obstruction (Biliary and Gastric Outlet): A Comprehensive Review with Clinical Scenarios

**DOI:** 10.3390/jcm13247731

**Published:** 2024-12-18

**Authors:** Giuseppe Dell’Anna, Rubino Nunziata, Claudia Delogu, Petra Porta, Maria Vittoria Grassini, Jahnvi Dhar, Rukaia Barà, Sarah Bencardino, Jacopo Fanizza, Francesco Vito Mandarino, Ernesto Fasulo, Alberto Barchi, Francesco Azzolini, Guglielmo Albertini Petroni, Jayanta Samanta, Antonio Facciorusso, Armando Dell’Anna, Lorenzo Fuccio, Sara Massironi, Alberto Malesci, Vito Annese, Nico Pagano, Gianfranco Donatelli, Silvio Danese

**Affiliations:** 1 Gastroenterology and Gastrointestinal Endoscopy Division, IRCCS San Raffaele Hospital, Via Olgettina 60, 20132 Milan, Italy; bara.rukaia@hsr.it (R.B.); bencardino.sarah@hsr.it (S.B.); fanizza.jacopo@hsr.it (J.F.); mandarino.francesco@hsr.it (F.V.M.); fasulo.ernesto@hsr.it (E.F.); barchi.alberto@hsr.it (A.B.); azzolini.francesco@hsr.it (F.A.); malesci.alberto@hsr.it (A.M.); danese.silvio@hsr.it (S.D.); 2 Gastroenterology and Gastrointestinal Endoscopy Division, IRCCS Policlinico San Donato, Piazza Edmondo Malan 2, 20097 San Donato Milanese, Italy; claudia.delogu01@universitadipavia.it (C.D.); guglielmo.albertinipetroni@grupposandonato.it (G.A.P.); vito.annese@grupposandonato.it (V.A.); 3 Department of Gastroenterology, Ospedale del Mare, ASL NA1 Centro, 80147 Naples, Italy; rubino.nunziata@gmail.com; 4 Istituti Clinici Scientifici Maugeri IRCCS, Gastroenterology Unit of Pavia Institute, 27100 Pavia, Italy; 5 Gastroenterology Unit, Department of Oncological and Specialty Medicine, Azienda Ospedaliero-Universitaria Maggiore della Carità, 28100 Novara, Italy; petraporta01@gmail.com (P.P.); mvittoriagrassini@gmail.com (M.V.G.); nico.pagano@maggioreosp.novara.it (N.P.); 6 Department of Gastroenterology and Hepatology, Punjab Institute of Liver and Biliary Sciences, Mohal 160062, India; jahnvi3012@gmail.com; 7 Department of Gastroenterology, Post Graduate Institute of Medical Education and Research, Chandigarh 160012, India; dj_samanta@yahoo.co.in; 8 Faculty of Medicine and Surgery, Vita-Salute San Raffaele University, Via Olgettina 56, 20132 Milan, Italy; sara.massironi@libero.it; 9 Faculty of Medicine and Surgery, University of Salento, Piazza Tancredi 7, 73100 Lecce, Italy; antonio.facciorusso@virgilio.it; 10 Digestive Endoscopy Unit, “Vito Fazzi” Hospital, Piazza Filippo Muratore 5, 73100 Lecce, Italy; armando.dellanna@alice.it; 11 Gastroenterology Unit, Department of Medical and Surgical Sciences, IRCCS Azienda Ospedaliero-Universitaria di Bologna, University of Bologna, 40100 Bologna, Italy; lorenzofuccio@gmail.com; 12 Unité d’Endoscopie Interventionnelle, Hôpital Privé des Peupliers, Ramsay Générale de Santé, 75013 Paris, France; donatelligianfranco@gmail.com; 13 Department of Clinical Medicine and Surgery, University of Naples “Federico II”, 80138 Naples, Italy

**Keywords:** malignant biliary obstruction, gastric outlet obstruction, double obstruction, EUS-guided biliary drainage, EUS-guided gastroenterostomy, EUS-guided choledochoduodenostomy, EUS-guided hepaticogastrostomy, EUS-guided antegrade stenting, EUS-guided gallbladder drainage

## Abstract

Endoscopic ultrasound (EUS)-guided interventions have revolutionized the management of malignant biliary obstruction (MBO) and gastric outlet obstruction (GOO), providing minimally invasive alternatives with improved outcomes. These procedures have significantly reduced the need for high-risk surgical interventions or percutaneous alternatives and have provided effective palliative care for patients with advanced gastrointestinal and bilio-pancreatic malignancies. EUS-guided biliary drainage (EUS-BD) techniques, including hepaticogastrostomy (EUS-HGS), choledochoduodenostomy (EUS-CDS), and antegrade stenting (EUS-AS), offer high technical and clinical success rates, with a good safety profile particularly when Endoscopic Retrograde Cholangiopancreatography (ERCP) is not feasible. EUS-HGS, which allows biliary drainage by trans-gastric route, is primarily used for proximal stenosis or in case of surgically altered anatomy; EUS-CDS with Lumen-Apposing Metal Stent (LAMS) for distal MBO (dMBO), EUS-AS as an alternative of EUS-HGS in the bridge-to-surgery scenario or when retrograde access is not possible and EUS-guided gallbladder drainage (EUS-GBD) with LAMS in case of dMBO with cystic duct patent without dilation of common bile duct (CDB). EUS-guided gastroenterostomy (EUS-GE) has already established its role as an effective alternative to surgical GE and enteral self-expandable metal stent, providing relief from GOO with fewer complications and faster recovery times. However, we do not yet have strong evidence on how to combine the different EUS-guided drainage techniques with EUS-GE in the setting of double obstruction. This comprehensive review aims to synthesize growing evidence on this topic by randomized controlled trials, cohort studies, and case series not only to summarize the efficacy, safety, and technical aspects of these procedures but also to propose a treatment algorithm based essentially on the anatomy and stage of the neoplasm to guide clinical decision-making, incorporating the principles of personalized medicine. This review also highlights the transformative impact of EUS-guided interventions on the treatment landscape for MBO and GOO. These techniques offer safer and more effective options than traditional approaches, with the potential for widespread clinical adoption. Further research is needed to refine these procedures, expand their applications, and improve patient care and quality of life.

## 1. Introduction

Malignant biliary obstruction (MBO) and gastric outlet obstruction (GOO) are severe complications associated with advanced gastrointestinal and bilio-pancreatic malignancies [1,2]. Individually, these conditions significantly impair patient quality of life and prognosis. They present a particularly formidable clinical challenge when they co-occur, forming a malignant double obstruction (MDO). Palliation of both obstructions is essential not only to alleviate symptoms and enhance the quality of life (QoL) but also to facilitate the initiation or continuation of systemic oncological therapies. Traditional endoscopic management of MDO, specifically Endoscopic Retrograde Cholangiopancreatography (ERCP) combined with enteral stenting (ES), often encounters technical challenges and is frequently associated with the need for multiple reinterventions [3,4].

Surgical bypass and percutaneous transhepatic biliary drainage (PTBD), while effective, are associated with high morbidity rates [5,6,7]. These limitations highlight the need for less invasive and more adaptable alternatives. In recent years, therapeutic endoscopic ultrasound (T-EUS) has emerged as a pivotal alternative in this setting [8,9]. T-EUS encompasses a range of techniques, including EUS-guided biliary drainage (EUS-BD) approaches such as hepaticogastrostomy (EUS-HGS), choledochoduodenostomy (EUS-CDS) and EUS-guided gallbladder drainage (EUS-GBD) and antegrade stenting (EUS-AS), and EUS-guided gastroenterostomy (EUS-GE).

These interventions have demonstrated high clinical efficacy and favorable safety profiles in palliating MDO [10,11,12]. However, the integration of T-EUS into managing MDO remains complex, with significant variability in clinical scenarios driven by factors such as tumor location, anatomical variations, and disease stage. The absence of robust comparative data on specific procedural combinations further complicates the development of standardized treatment strategies. This review aims to critically examine the current role of T-EUS in managing malignant double obstruction. By synthesizing available evidence and integrating findings from recent studies, we propose a treatment algorithm tailored to the unique challenges of this condition, with a focus on optimizing patient outcomes through personalized, evidence-based approaches.

## 2. Materials and Methods

In our review, we searched PubMed, PubMed Central (PMC), and Medline, including only English-written articles published by the end of November 2024. We implemented a comprehensive search strategy using strings including following terms: the use of strings including “biliary obstruction”, “gastric outlet obstruction”, “malignant”, “EUS”, “endoscopic ultrasound”, “endoscopy”, “endoscopic stenting”, “gastrojejunostomy”, “gastroenterostomy” “gastroenteroanastomosis”, “biliary drainage”, “choledocoduodenostomy”, “hepaticogastrostomy”, “antegrade stenting”, and “gallbladder drainage”. Additionally, we manually searched the references of the included studies and relevant reviews to identify any additional suitable publications.

## 3. EUS-Guided Biliary Drainage

EUS-BD procedures allow obtaining a decompression of the biliary system through a connection between the bile ducts and the upper GI lumen (stomach, duodenum, jejunum). According to the biliary access site, we can distinguish two main types of EUS-BD: intrahepatic and extrahepatic procedures [12]. The intrahepatic group involves EUS-HGS, EUS-AS, and EUS-RVS. The extrahepatic group includes EUS-CDS, EUS-GBD, and EUS-RVS. Concerning EUS-RVS, this procedure should be considered an EUS-assisted and not an EUS-guided one, according to the ESGE technical review [9,13].

Over the years, EUS-BD has gained significant recognition, supported by national and international guidelines that recommend it as the primary alternative in ERCP failure cases for treating malignant biliary strictures. Additionally, it can be considered a first-line treatment in the appropriate setting, specifically in referral centers, for patients with unresectable disease [13,14,15].

A recent meta-analysis by Ginnaram SR et al. compared outcomes of EUS-BD and ERCP as first-line approaches for malignant biliary obstruction [16]. The analysis included seven comparative studies (four RCTs and three retrospective studies) with a total of 1245 patients. The results suggested a potentially higher TS rate for EUS-BD compared to ERCP, with a common effects model (CEM) odds ratio (OR) of 1.7 (95% CI, 1.00–3.14) and a random effects model (REM) OR of 1.37 (95% CI, 0.51–3.70). No significant differences were observed in CS rates, with a CEM OR of 0.79 (95% CI, 0.43–1.43) and an REM OR of 0.80 (95% CI, 0.43–1.47), or in overall adverse events (AEs) rate, with a CEM OR of 0.80 (95% CI, 0.53–1.20) and an REM OR of 0.81 (95% CI, 0.51–1.27) [16]. EUS-BD was associated with a significantly lower rate of post-procedural acute pancreatitis compared to ERCP, with a CEM OR of 0.09 (95% CI, 0.02–0.34) and an REM OR of 0.10 (95% CI, 0.03–0.39). Additionally, EUS-BD appeared superior in terms of reintervention rates, with a CEM OR of 0.58 (95% CI, 0.36–0.92) and an REM OR of 0.64 (95% CI, 0.33–1.24). No substantial differences were found in other secondary outcomes, such as procedural time, hospital stay, and 30-day mortality [16].

Lauri G et al. recently published a comparative network meta-analysis that included only RCTs comparing the feasibility, efficacy, and safety of EUS-CDS with LAMS, EUS-CDS with SEMS, EUS-HGS, ERCP, and PTBD as primary techniques for biliary drainage in distal malignant biliary obstruction [17]. The final analysis included six RCTs with a total of 583 patients. EUS-CDS with LAMS demonstrated a significantly higher TS rate compared to EUS-CDS with SEMS (RR 1.21, 95% CI 1.07–1.37) and ERCP (RR 1.17, 95% CI 1.07–1.28). It also showed a higher CS rate compared to ERCP (RR 1.12, 95% CI 1.01–1.25). PTBD ranked as the least safe procedure (SUCRA score = 0.18), while EUS-CDS with LAMS was identified as the best option for operative time (SUCRA score = 0.83) [17].

Khoury T et al. published a systematic review and meta-analysis focusing exclusively on RCTs comparing EUS-BD and ERCP as primary drainage techniques for malignant, non-surgical distal biliary obstruction [18]. The analysis included five RCTs with a total of 519 patients. No significant differences were observed between EUS-BD and ERCP in terms of pooled TS (TS; *p* = 0.27), CS (CS; *p* = 0.45), 1-year stent patency (*p* = 0.17), or safety (*p* = 0.55). However, EUS-BD demonstrated superiority in terms of a lower reintervention rate compared to ERCP (RR 0.58, 95% CI 0.37–0.90; *p* = 0.01). EUS-BD was also associated with significantly shorter procedural time compared to ERCP (standardized mean difference: −2.36 min [−2.68 to −2.05]; *p* < 0.001). Subgroup analysis further revealed that EUS-BD with LAMS had a significantly higher TS rate compared to ERCP (RR 1.17, 95% CI 1.01–1.35; *p* = 0.03) [18].

In this section of our review, we discuss the main technical aspects of EUS-BD, its efficacy, safety profile, and the indications for each technique, tailored to the location of the obstruction (distal or hilar) and patient characteristics. Currently, no consensus exists on the superior technique, emphasizing the need for an individualized approach.

### 3.1. EUS-Guided Choledochoduodenostomy (EUS-CDS)

**Figure 1:**
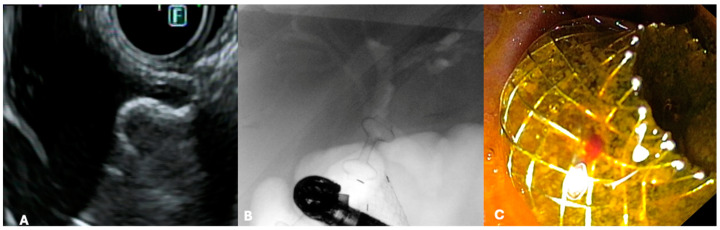
Endoscopic Ultrasound-Guided Choledochoduodenostomy (EUS-CDS). (**A**) EUS view of the distal flange of electrocautery-enhanced Lumen-Apposing Metal Stent (ec-LAMS) released into the common bile duct. (**B**) Fluoroscopy view of EUS-CDS with aereobilia confirming the correct placement. (**C**) Endoscopic view of the proximal flange of ec-LAMS correctly deployed into the duodenal bulb lumen. The copyright of the image belongs to the authors.

EUS-CDS consists of creating a bilio-digestive anastomosis between the common bile duct (CBD) and the duodenum [12] (Figure 1).

Under EUS guidance, from the duodenal bulb, the dilated CBD (>12 for expert operators, >15 mm for non-expert operators) was identified with the EUS scope in a “long” position, with the tip of the endoscope in the direction of the hepatic hilum [19]. At this stage, a bilio-digestive anastomosis is created by advancing an electrocautery-enhanced LAMS (ec-LAMS) directly into the CBD lumen under EUS guidance. While EUS-CDS can theoretically be performed without fluoroscopic assistance, in cases of a small-diameter CBD, the free-hand technique may be replaced with the over-the-wire technique [20]. This approach involves initially puncturing the CBD, preferably with a 19-gauge FNB needle, followed by the advancement of a guidewire into the CBD and performing a cholangiogram to confirm accurate access. The ec-LAMS catheter can then be advanced over the wire into the CBD, allowing for precise deployment of the LAMS. Indications from international societies (ESGE, ASGE) have evolved during the last couple of years. In 2022, the ESGE guidelines recommended EUS-CDS over EUS-HGS as the preferred technique for managing dMBO, citing a lower rate of adverse events (AEs) associated with EUS-CDS [13]. However, subsequent evidence has provided conflicting data regarding the safety profiles of these two procedures. A meta-analysis and systematic review by Yamazaki H et al., published in 2023, analyzed 18 studies encompassing 972 patients and found no significant difference in AEs between EUS-CDS and EUS-HGS (OR 1.39; 95% CI 1.00–1.93) [21]. In contrast, a newer meta-analysis by Rizqiansyah SY et al., published a year later, included 11 comparative studies (both controlled and uncontrolled) with a total of 537 patients and reported a significantly higher AE rate in the EUS-HGS group compared to the EUS-CDS group [22]. Furthermore, growing evidence showed that EUS-CDS may be associated with a risk of ascending cholangitis in the case of concomitant GOO [23,24]. In this view, the ASGE guideline suggested EUS-HGS over EUS-CDS in case of duodenal malignant infiltration, pre-existing ES, or GOO proximal to the pylorus [15].

Recently, two international multicenter RCTs comparing EUS-CDS with LAMS and ERCP as a first-line strategy for biliary drainage in dMBO were published [25,26]. The DRA-MBO trial by Teoh AYB et al. enrolled 155 patients with unresectable malignant biliary obstruction (79 EUS-CDS, 76 ERCP) [26]. The primary outcome was the 1-year stent patency rate, which showed no significant difference between the two groups (EUS-CDS: 91.1% vs. ERCP: 88.1%, *p* = 0.52). However, the EUS-CDS group demonstrated a significantly higher technical success (TS) rate compared to the ERCP group (EUS-CDS: 96.2% vs. ERCP: 76.3%, *p* < 0.001) and a similar CS (defined as >30% drop in bilirubin levels within 5 days) rate (EUS-CDS: 93.7% vs. ERCP: 90.8%, *p* = 0.559). Additionally, EUS-CDS was associated with a significantly shorter procedural time (EUS-CDS: 10 min [IQR 5.75–18] vs. ERCP: 25 min [IQR 14–40], *p* < 0.001). Regarding safety, both procedures had comparable AE rates [26]. The ELEMENT trial by Chen YI et al. included 144 patients with dMBO (73 EUS-CDS, 71 ERCP) [25]. Unlike the DRA-MBO trial, this RCT included patients with borderline resectable/locally advanced disease (38.9%) and unresectable disease (61.1%). The primary endpoint was the 1-year stent dysfunction rate, which was similar between the two groups (EUS-CDS: 9.6% vs. ERCP: 9.9%, *p* = 0.96). The TS rate also showed no significant difference, with EUS-CDS achieving 90.4% (95% CI 81.5–95.3%) compared to ERCP at 83.1% (95% CI 72.7–90.1%), yielding a risk difference of 7.3% (95% CI −4.0–18.8%), confirming non-inferiority. The two groups also showed no differences in pancreaticoduodenectomy oncological outcomes, AEs, or quality of life [25].

Li JS et al. recently published a systematic review and meta-analysis evaluating the safety profile of EUS-CDS with LAMS, including 21 studies with a total of 1438 patients [27]. The primary endpoint was the incidence of overall and specific adverse events (AEs). The overall AE rate was 20.1% (95% CI 16.0–24.9). Early and long-term AEs were reported in 10.6% (95% CI 7.9–14.2) and 11.2% (95% CI 8.2–15.2) of cases, respectively. Cholangitis was the most common AE, with a pooled incidence of 6.1% (95% CI 3.7–10.1), followed by stent maldeployment at 5.7% (95% CI 3.4–9.4). The pooled incidences of stent migration and bleeding were 2.4% (95% CI 1.4–4.1) and 2.3% (95% CI 1.4–3.7), respectively. Other AEs included bile leak (2.7%, 95% CI 1.2–5.8), peritonitis (2.7%, 95% CI 1.2–5.8), perforation (2.4%, 95% CI 1.3–4.4), cholecystitis (2.0%, 95% CI 0.8–4.8), and pancreatitis (4.0%, 95% CI 0.9–15.3). Regarding stent dysfunction and reintervention, the pooled incidences were 10.5% (95% CI 7.5–14.4) and 12.1% (95% CI 9.3–15.7), respectively [27].

The increase in survival of patients with bilio-pancreatic diseases, in particular pancreatic cancer, due to the introduction of personalized therapies has led to an increase in the number of patients with long-term EUS-CDS, raising the issue of diagnosis and treatment of long-term dysfunctions [28,29].

Vanella G et al. conducted a large retrospective international multicenter study involving 93 patients who underwent EUS-CDS to evaluate stent dysfunction and identify associated factors [28]. The study reported technical success (TS) and clinical success (CS) (defined as a ≥50% bilirubin decrease during follow-up) rates of 97.8% and 93.4%, respectively, with an adverse event (AE) rate of 9.7% (mostly mild to moderate). Stent dysfunction, defined as the new onset or worsening of bilirubin levels and/or the occurrence of cholangitis in patients who initially achieved CS, was observed in 31.8% of patients after a mean of 166 days (95% CI 91–241), with a mean dysfunction-free survival (DFS) of 394 days (95% CI 307–482). Estimated DFS at 6 months and 12 months was 75% and 52%, respectively. The duodenal invasion was identified as the only independent factor for dysfunction (hazard ratio 2.7, 95% CI 1.1–6.8). The authors introduced a comprehensive classification system, the L.A.M.S. Classification, for both dysfunction types and rescue procedures. Dysfunction types were categorized as follows: (1) Sump Syndrome, (2a) Stone/Sludge Impaction, (2b) Food Impaction, (3a) LAMS Invasion/Compression on the Biliary Side, (3b) LAMS Invasion/Compression on the Duodenal Side, (4) LAMS Migration, and (5) GOO. Rescue procedures were classified as follows: (A) Coaxial DPPS, (B) Balloon/Basket Swipes, (C) Through-the-LAMS Biliary SEMS, (D1) Transpapillary SEMS (retrograde via standard cannulation), (D2) Transpapillary SEMS (via LAMS for rendezvous), (D3) Transpapillary SEMS (via LAMS for antegrade stenting), (E) Over-the-Wire LAMS Exchange, (F) Resolution of GOO, (G1) Redo EUS-CDS, (G2) EUS-HGS, and (G3) PTBD. The most common dysfunction causes in this series were stone and sludge impaction (33.3%) and GOO (25.9%). The most frequently performed rescue procedure was coaxial DPPS placement (44.4%) [28].

New technical advancements have been proposed in recent years to reduce dysfunction rates and improve long-term outcomes.

Garcia-Sumalla A et al., in their international multicenter RCT, compared the outcomes of EUS-CDS with LAMS, with or without through-the-LAMS double pigtail plastic stents (DPPSs) [30,31]. The primary endpoint was the rate of recurrent biliary obstruction (RBO). In contrast, secondary endpoints included TS, CS (defined as a bilirubin reduction > 50% within 14 days), and AE rates. A total of 84 patients were enrolled: 44 in the LAMS group and 40 in the LAMS-DPPS group. The rate of RBO was significantly higher in the LAMS group compared to the LAMS-DPPS group (31.8% vs. 12.5%; OR 3.26 [95% CI 1.05–10.12], *p* = 0.04). Conversely, the operative time was significantly longer in the LAMS-DPPS group (30 vs. 20 min, *p* = 0.02). The LAMS group showed higher rates of biliary reinterventions (22.7% vs. 12.5%, *p* = 0.26) and a longer time-to-RBO (51.5 vs. 155.5 days, *p* = 0.10), though these differences were not statistically significant. However, the AE rate was significantly higher in the LAMS-DPPS group (4.5% vs. 20%, *p* = 0.04) [30,31].

Similarly, Fritzsche JA et al. [32] recently published a prospective single-center pilot study (SCORPION-IIp study) involving 27 patients with distal malignant biliary obstruction (dMBO) without GOO. The patients were treated with EUS-CDS using LAMS combined with a through-the-LAMS FC-SEMS to align the CBD axis with the descending duodenum, aiming to reduce stent dysfunction, which was the study’s primary outcome. The TS rates for LAMS placement and through-the-LAMS FC-SEMS placement were 89% (24/27) and 83% (20/24), respectively. In the four cases of technical failure, a through-the-LAMS DPPS was placed instead of an FC-SEMS. The overall CS (defined as at least a 50% decrease in bilirubin and/or relief of symptoms without reintervention within 30 days) rate was 90%, as 2 out of 20 patients required stent revision. After a median follow-up of 152 days (IQR 38–180), no stent dysfunction was observed in the LAMS+FC-SEMS group [32].

### 3.2. EUS-Guided Hepaticogastrostomy (EUS-HGS)

Among EUS-BD procedures utilizing the intrahepatic route, EUS-HGS enables direct biliary drainage from the left bile ducts to the stomach [12]. This approach is beneficial for patients with an inaccessible papilla (e.g., due to malignant duodenal infiltration) or those with surgically altered anatomy (SAA) [33].

**Figure 2:**
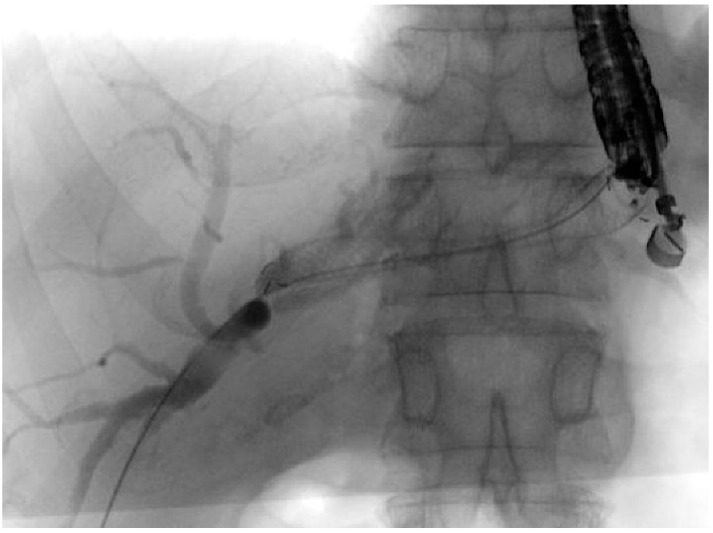
Endoscopic Ultrasound-Guided-Hepaticogastrostomy (EUS-HGS) with partially covered metal stent (PC-SEMS). The copyright of the image belongs to the authors.

EUS-HGS is likely the most technically demanding EUS-BD procedure, primarily due to the required familiarity with multiple devices and the need for frequent device exchanges, as well as the limited operative space within the intrahepatic bile ducts [33]. During the last years, there has been a growing interest in technical standardization and in developing new devices, particularly dedicated SEMS (dedicated ec-PC-SEMS), to improve the safety profile of EUS-HGS (Figure 2) [33,34].

The first technical step in EUS-HGS is selecting the puncture site, typically performed under EUS guidance in segment 2 (B2) or segment 3 (B3) of the left hepatic lobe [33]. The bile duct should have a minimum diameter of >2 mm for expert operators or >5 mm for non-experts. Puncture is usually performed using a 19-gauge FNB needle. Following puncture, contrast injection through the needle is essential to perform a cholangiogram to confirm proper positioning and proceed. Some operators also aspirate bile to prevent pneumobilia and ensure entry into the bile duct. Next, a guidewire is advanced into the bile duct and directed distally, ideally through the papilla. The hepatic–gastric tract is then created by advancing an electrocautery-enhanced cystotome (typically 6Fr in caliber) from the stomach into the bile duct. Alternatively, pneumatic dilation of the tract can be performed. Finally, a dedicated PC-SEMS (variable length from 80 to 100 mm) is advanced over the guidewire, with the uncovered distal portion (approximately 30% of the entire length) deployed in the bile duct and the proximal fully covered portion (approximately 70% of the entire length) in the gastric lumen [33].

According to the 2022 ESGE guidelines, EUS-HGS is indicated for cases of malignant, inoperable hilar biliary obstruction with left bile duct dilation when ERCP has failed [13]. The ASGE guidelines, as previously mentioned, also recommend EUS-HGS for distal malignant biliary obstruction (dMBO) with concomitant duodenal malignant infiltration and/or the presence of an enteral stent (ES) [15]. Technical contraindications for EUS-HGS include massive ascites, as the increased distance between the liver and gastric wall can elevate the risk of adverse events such as bile leakage, peritonitis, bleeding, and SEMS migration. Other contraindications are neoplastic infiltration of the gastric wall, portal hypertension, and the presence of perigastric collateral vessels [9,33].

The largest meta-analysis to date evaluating the efficacy and safety of EUS-HGS was recently published by Moond V et al., encompassing 44 studies (25 retrospective and 19 prospective) with a total of 1576 patients [35]. EUS-HGS demonstrated a pooled TS rate of 94.4% (95% CI 92.4–95.9%; I^2^ = 0%) and a pooled CS rate of 88.6% (95% CI 83.7–92.2%; I^2^ = 0%). Regarding safety, the pooled AE rate for EUS-HGS was 5.8% (95% CI 4.2–8.1%; I^2^ = 0%). The pooled rates of AEs by severity were as follows: mild AEs at 5.8% (95% CI 4.2–8.1%; I^2^ = 0%), moderate AEs at 12.1% (95% CI 9.1–15.8%; I^2^ = 16%), severe AEs at 4.2% (95% CI 3.0–5.7%; I^2^ = 61%), and fatal AEs at 3.2% (95% CI 1.9–5.4%; I^2^ = 62%). Stent migration was the most common AE, occurring in 5.3% of cases, followed by cholangitis (4.9%), bleeding (4.9%), bile leak or bile peritonitis (4.9%), stent occlusion (4.2%), and perforation (3.5%) [35].

Binda C et al. published a systematic review, meta-analysis, and meta-regression analysis examining the features and outcomes of EUS-HGS during the same period [36]. The study’s primary outcome was the TS rate, with secondary outcomes including CS and AEs. A total of 33 studies comprising 1644 patients were included, with MBO being the primary underlying cause in 99.6% of cases. The main indications for EUS-HGS were duodenal or papillary infiltration (34.8%), SAA (18.4%), and hilar biliary obstruction (16%). Consistent with the findings of Moond V et al., EUS-HGS demonstrated a pooled TS rate of 97.7% (95% CI 96.1–99.0%; I^2^ = 0%) and an intention-to-treat CS rate of 88.1% (95% CI 84.7–91.2%; I^2^ = 33.9%) [35]. AEs occurred in 12.0% of cases (95% CI 9.8–14.5%; I^2^ = 20.4%), with cholangitis/sepsis (2.8%) and bleeding (2.3%) being the most common complications. Interesting insights were gained from a univariable meta-regression analysis to identify potential primary and secondary outcome modifiers. TS was found to improve significantly in centers with more experience (>4 cases/year; OR 2.12, 95% CI 1.23–3.67; *p* = 0.007), in the presence of duodenal invasion (OR 6.56, 95% CI 1.18–36.4; *p* = 0.03), and when dedicated HGS SEMS were used (OR 2.22, 95% CI 1.24–3.99; *p* = 0.007). For secondary outcomes, center experience (>4 cases/year) was the only significant modifier associated with increased CS (OR 1.47, 95% CI 1.01–2.13; *p* = 0.04). Additionally, the used stents were the only variable associated with a reduced risk of AEs (OR 0.62, 95% CI 0.41–0.95; *p* = 0.03) [36].

The long-term outcomes of EUS-HGS were evaluated in a large multicenter retrospective study by Hedjoudje A et al., which included 198 patients with a median overall survival of 144 days (IQR 108–211) [37]. Most patients had pancreatic cancer (49.5%) or cholangiocarcinoma (14.6%), with 57.3% presenting with metastatic disease at diagnosis. MBO was proximal/hilar in 68.4% of cases and distal in 27.6%. After a median follow-up of 56 days (IQR 21–187), the overall AE rate was 33%. Among major AEs, infection (18.3%) and abdominal pain (12.1%) were the most common, followed by bleeding (6.1%), peritonitis (5.6%), cholangitis (4.5%), bilioma (3.2%), and cholecystitis (1.1%). During the study period, 38 cases of RBO (19.1%) were reported. Kaplan–Meier analysis showed stent patency probabilities of 88.9% (95% CI 84.1–93.9) at 30 days, 82.2% (95% CI 75.8–89.1) at 90 days, 69.5% (95% CI 60.2–80.1) at 180 days, and 61.7% (95% CI 50.7–75.2) at 365 days. Univariate and multivariate Cox regression analyses identified factors associated with stent patency. PC-SEMS were associated with a significantly lower risk of RBO in both univariate (OR 0.42, 95% CI 0.21–0.82, *p* = 0.012) and multivariate analyses (OR 0.47, 95% CI 0.24–0.95, *p* = 0.034), compared to FC-SEMS. Additionally, patients with dMBO demonstrated significantly better stent patency in univariate (HR 0.13, *p* = 0.066) and multivariate analyses (HR 0.06, *p* = 0.031). The most common cause of RBO was tumor ingrowth (36.8%), followed by food impaction and sludge formation (21.1%), hyperplasia at the uncovered portion of the stent (15.8%), and bleeding or clots (7.9%). Endoscopic management was performed in 22 cases (57.9%) and was successful in 90.9%, with stent-in-stent placement being the most frequently performed procedure [37].

A technical variation of EUS-HGS involves advancing the guidewire transpapillary to place a transpapillary SEMS in an antegrade fashion, effectively combining EUS-HGS with EUS-AS. This approach, referred to as EUS-HGS+EUS-AS, was first described in 2017 by Imai et al. [38]. Its objective is to minimize the risk of bile leakage and create dual drainage routes (trans-gastric and transpapillary) to ensure biliary drainage in cases of EUS-HGS dysfunction.

Paraskevopoulos P et al. recently published a systematic review and meta-analysis comparing EUS-HGS (HGS group) outcomes and EUS-HGS+EUS-AS (HGAS group) [39]. The analysis included 26 studies with 1083 patients—788 in the HGS group and 295 in the HGAS group. The HGS group demonstrated a significantly higher pooled TS rate compared to the HGAS group [94% (95% CI 92–96%) for HGS vs. 89% (95% CI 83–93%) for HGAS; *p* = 0.00273]. Conversely, the pooled CS rate was significantly higher in the HGAS group [94% (95% CI 89–97%) for HGAS vs. 88% (95% CI 84–91%) for HGS; *p* = 0.0367]. The HGAS group also exhibited a lower pooled AE rate compared to the HGS group [14% (95% CI 9–20%) for HGAS vs. 20% (95% CI 16–25%) for HGS; *p* = 0.0728]. Regarding reintervention rates, the HGAS group showed a significantly lower rate, with an odds ratio of 0.37 (95% CI 0.27–0.52; *p* = 0.006), favoring the dual-drainage approach [39].

### 3.3. EUS-Guided Antegrade Stenting (EUS-AS)

EUS-AS shares several initial technical steps with EUS-HGS, including carefully selecting a puncture site under EUS guidance, typically in a sufficiently dilated intrahepatic bile duct from B2 or B3. Some experts recommend choosing B2 bile ducts due to their straighter course toward the major papilla than B3 ducts [9,40]. As with EUS-HGS, liver puncture is generally performed using a 19-gauge FNB needle. Following contrast injection to confirm bile duct access, a guidewire is advanced through the stenosis in the CBD and toward the papilla into the duodenal lumen. At this point, the operator has several technical options. The SEMS release catheter can be advanced directly through the stenosis and the stent deployed across it. If the stenosis is particularly tight, pneumatic dilation can be performed first to facilitate the passage of the SEMS catheter. To accurately assess the stenosis and select the appropriate SEMS size, a retrieval balloon can be advanced to the end of the stenosis, inflated, and a cholangiogram performed. The final step involves releasing the SEMS in an antegrade fashion [40].

A lower amount of evidence is now available specifically on EUS-AS compared to other EUS-BD procedures (e.g., EUS-CDS, EUS-HGS, EUS-GBD).

The first retrospective series evaluating the role of EUS-AS in managing MBO after failed ERCP was published by So H et al. in 2021, including 25 patients with unresectable disease [41]. GOO was the primary indication for EUS-BD (72%), followed by SAA (28%). Among patients with GOO, 11/18 (61.1%) had a type II stenosis, and 7/18 (38.9%) had a type I stenosis, with seven undergoing ES placement. EUS-AS was performed after ES placement in four patients and vice versa in three patients. Advanced gastric cancer was the most common cause of MBO (44%), followed by pancreatic cancer (32%).

In all cases, the authors used an 8 mm diameter FC-SEMS measuring 11–13 cm in length. EUS-AS demonstrated a TS rate of 96%, with one technical failure due to the guidewire’s inability to pass a tight stenosis caused by pancreatic cancer; this patient subsequently underwent EUS-HGS. CS (defined as a decrease in bilirubin level to normal or to less than a quarter of the pretreatment value within the first month) was achieved in 84% of cases, with clinical failures attributed to advanced neoplastic disease. During the study period, six AEs (25%) related to the EUS-AS procedure were observed: four cases of acute pancreatitis and two cases of FC-SEMS migration. Two patients required PTBD due to FC-SEMS obstruction, with a median stent patency time of 9.40 months (95% CI 7.96–NA). The authors also compared the outcomes of EUS-AS with those of a similar cohort of patients undergoing conventional EUS-BD procedures (EUS-CDS and EUS-HGS) during the same period. No differences were noted in TS, CS, or AE rates. However, EUS-AS was associated with a lower reintervention rate (HR 0.242, 95% CI 0.057–1.035; *p* = 0.056) and significantly longer median stent patency compared to the conventional EUS-BD group (HR 0.236, 95% CI 0.071–0.777; *p* = 0.018) [41].

In 2022, Sundaram A et al. published a larger retrospective study on 54 patients with MBO treated with EUS-AS for either preoperative biliary drainage (37%) or palliative drainage (62.9%) [42]. The stenosis was distal in 64.8% of cases and proximal in 35.1%. Most patients had pancreatic cancer (44.4%), followed by ampullary carcinoma (11.1%) and gastric cancer (7.3%). The primary indication for EUS-AS was an inaccessible papilla due to concomitant gastro-duodenal malignant stenosis (75.9%), followed by failed cannulation (18.5%) and SAA (5.5%). The site of EUS-guided bile duct puncture was evenly distributed between segments B2 and B3, using either a 19-gauge or 22-gauge FNB needle. The median size of the punctured intrahepatic bile ducts was 6.1 mm (IQR 5.5–7.1). In most cases, an uncovered SEMS (uc-SEMS) was placed (95.8%), with a 60 mm length SEMS preferred in 59.6% of cases. TS was achieved in 88.7% of cases, with a CS (defined as a decrease in bilirubin by >50% at 2 weeks or normalization at 4 weeks) rate of 95.7%. The overall AE rate was 7.4%, including two cases of self-limiting pneumoperitoneum without clinical relevance, one case of post-procedural cholecystitis treated conservatively, and one case of mild acute pancreatitis. In terms of RBO, only two patients in the palliative group required biliary reinterventions. Among the preoperative biliary drainage group, 10 patients (50%) proceeded to surgery, including 5 who underwent neoadjuvant chemotherapy (median time to surgery of 155 days, IQR 133–194). Two patients developed non-biliary grade III/IV complications according to the Clavien–Dindo classification (pancreatic fistula and bleeding in the surgical bed) [42].

Shen Y et al. recently published a retrospective comparative study evaluating EUS-AS and EUS-BD with transluminal stent implantation (EUS-TLS) in 82 patients with unresectable dMBO following failed ERCP [43]. The study included 45 patients in the EUS-TLS group (comprising EUS-CDS and EUS-HGS) and 37 in the EUS-AS group. Baseline characteristics were similar between the groups, with pancreatic cancer being the most common underlying disease (26.7% EUS-TLS, 29.7% EUS-AS). GOO was the primary indication for EUS-BD in both groups (55.5% EUS-TLS, 51.4% EUS-AS), followed by SAA (24.4% EUS-TLS, 27.0% EUS-AS). For GOO management, 20.0% of patients in the EUS-TLS group and 10.8% in the EUS-AS group underwent EUS-GE, while 17.8% and 10.8%, respectively, underwent ES placement. The EUS-TLS group demonstrated a significantly higher TS rate compared to the EUS-AS group (97.8% vs. 81.1%; *p* = 0.031). In the EUS-TLS group, technical failures were attributed to LAMS misdeployment during EUS-CDS, whereas in the EUS-AS group, all seven technical failures were due to guidewire failure to pass the distal stenosis. The EUS-AS group exhibited a higher CS (defined as a decrease in total bilirubin by at least 50% within 14 days of EUS-BD compared with the pre-procedural level) rate than the EUS-TLS group (90.0% vs. 84.1%; *p* = 0.701) and a better safety profile, with an overall AEs of 13.5% (three cases of bile peritonitis, two cases of cholangitis) compared to 20.0% in the EUS-TLS group (five cases of bile peritonitis, three cases of cholangitis, one case of bleeding). No significant differences were observed between the groups regarding rates of stent migration (*p* = 0.821), stent dysfunction (*p* = 0.346), or need for reintervention (*p* = 0.845). For biliary reinterventions, one patient in the EUS-TLS group underwent PTBD, one underwent ERCP, and six underwent SEMS exchange via the bilio-digestive fistula. In the EUS-AS group, four patients required PTBD, and two underwent ERCP. Kaplan–Meier analysis revealed a significantly longer median time to stent dysfunction in the EUS-AS group (81 days) compared to the EUS-TLS group (53 days; *p* = 0.017) [43].

### 3.4. EUS-Guided Gallbladder Drainage (EUS-GBD)

EUS-guided gallbladder drainage (EUS-GBD) involves creating communication between the gallbladder and the gastric or duodenal lumen [9]. Under EUS guidance, the distended gallbladder is visualized, preferably via the gastric antrum or the duodenal bulb. Using either a free-hand or over-the-wire technique, the ec-LAMS catheter is advanced into the gallbladder lumen, and the LAMS is deployed under EUS and endoscopic guidance. In many cases, a DPPS is placed to prevent food or stone impaction; however, current evidence does not support its routine use. Similarly, no randomized comparative data exist favoring the gastric or duodenal route. Theoretically, trans-gastric drainage may pose a higher risk of food impaction and buried LAMS syndrome compared to trans-duodenal drainage [9]. In the bridge-to-surgery scenario, multidisciplinary discussions are crucial [44]. For patients with acute cholecystitis who are currently unfit for surgery but may be surgical candidates in the future, the gastric route is preferred due to the greater feasibility of suturing gastric access during surgery. Conversely, for biliary drainage in patients with dMBO undergoing neoadjuvant chemotherapy and potential future surgery (e.g., pancreaticoduodenectomy), the duodenal route is preferred, as both the gallbladder and duodenum are typically removed during the procedure [44].

EUS-GBD has an established role in managing acute cholecystitis in patients unfit for surgery, demonstrating superiority over percutaneous gallbladder drainage (PTGBD) and endoscopic transpapillary drainage in terms of long-term outcomes, feasibility, and lower associated morbidity [13,45,46].

International societies regard EUS-GBD as a rescue technique for biliary drainage in cases of dMBO, particularly after failed ERCP and when EUS-CDS is not feasible due to technical challenges [13,14,15].

Binda C et al. recently published a large retrospective multicenter study involving 48 patients with dMBO who underwent EUS-GBD as a rescue treatment [47]. Pancreatic adenocarcinoma was the most common underlying cause of dMBO (85.4%), followed by cholangiocarcinoma (4.2%). The primary reasons for failed ERCP were major papilla infiltration (33.3%) and malignant duodenal obstruction (29.2%). The free-hand technique was preferred in most cases (93.8%). A 10 × 10 mm ec-LAMS was used in 70.8% of cases, and an 8 × 8 mm ec-LAMS in 20.8%. The trans-gastric route was employed in 58.3% of patients, while the trans-duodenal route was used in 41.7%. CS, defined as a ≥50% reduction in bilirubin levels within two weeks of the procedure, was achieved in 81.3% of patients (39/48). Clinical failure cases were managed with PTBD. A total of five AEs (10.4%) were reported, including three intraprocedural and two delayed AEs (>15 days). According to the ASGE Lexicon classification, three AEs were moderate, and two were mild. Among the intraprocedural AEs, two cases involved bleeding (only one required endoscopic hemostasis), and one case involved LAMS dislodgment, which was managed with PTBD. Long-term AEs included LAMS occlusion and buried LAMS, both successfully treated using the LAMS-in-LAMS technique. The authors also performed a univariate analysis, but no variables were significantly associated with clinical success [47].

Mangiavillano B. et al. conducted a prospective multicenter study evaluating EUS-GBD as a first-line approach for biliary drainage in 37 patients with dMBO [48]. CS was defined as a reduction in bilirubin levels of >15% within 24 h and >50% within 14 days after EUS-GBD. Both TS and CS rates were 100%. After a median overall survival of 4 months (95% CI 1–8), the AE rate was 10.8%, including one case of bleeding, one case of food impaction, and two cases of cystic duct obstruction caused by disease progression [48].

The only systematic review and meta-analysis assessing the efficacy and safety of EUS-GBD as a rescue strategy for biliary drainage was published by Kamal F et al. in 2022 [49]. This analysis included five studies with a total of 104 patients. The pooled CS rate was 85% (95% CI 76–91%), while the pooled AE rate was 13% (95% CI 7–21%). Regarding long-term outcomes, the pooled rate of stent dysfunction requiring repeat biliary procedures was 9% (95% CI 4–21%) [49]. The more recent systematic review by McDonagh et al. showed similar results [50].

Debourdeau A et al. recently published the GALLBLADEUS study, a multicenter retrospective analysis comparing the efficacy and safety of EUS-CDS (37 patients) and EUS-GBD (41 patients) in 78 individuals with dMBO following failed ERCP [51]. Most patients in both groups had metastatic pancreatic adenocarcinoma. In the EUS-GBD group, difficult biliary cannulation was the leading cause of ERCP failure (56.1%), whereas in the EUS-CDS group, duodenal stenosis accounted for most failures (37.8%). All EUS-CDS procedures were performed using 6 × 8 mm ec-LAMS, while the EUS-GBD group predominantly used 15 × 10 mm ec-LAMS (63.4%). The CBD diameter was significantly larger in the EUS-CDS group compared to the EUS-GBD group (16 ± 5 mm vs. 13 ± 3 mm, *p* < 0.001). No significant differences were observed in TS (100% EUS-GBD vs. 94.6% EUS-CDS, *p* = 0.132) or CS [defined by a >50% decrease in total bilirubin levels at day 7 or normalization at day 28 (<48 μmol/L)] (87.8% EUS-GBD vs. 89.2% EUS-CDS, *p* = 0.8) between the two groups. Although the EUS-GBD group exhibited a slower reduction in bilirubin levels compared to the EUS-CDS group (*p* = 0.039), the proportion of patients who initiated neoadjuvant chemotherapy was similar (47.37% EUS-GBD vs. 58.33% EUS-CDS, *p* = 0.477). The safety profiles of the two techniques were comparable, with AE rates of 9.76% in the EUS-GBD group and 24.32% in the EUS-CDS group (*p* = 0.128) and LAMS obstruction rates of 28.57% (EUS-GBD) vs. 38.46% (EUS-CDS, *p* = 0.083). However, late AE rates (occurring >24 h post-procedure) were significantly higher in the EUS-CDS group (21.6%) compared to the EUS-GBD group (7.3%, *p* = 0.042). Similarly, LAMS dysfunction rates, defined per the L.A.M.S. Classification, were higher in the EUS-CDS group (21.62%) than in the EUS-GBD group (12.2%), although this difference was not statistically significant (*p* = 0.364) [28]. A subgroup analysis was conducted for patients with concomitant GOO due to duodenal stenosis. No significant differences were noted between the groups in CS rates (88.89% EUS-CDS vs. 88.24% EUS-GBD, *p* > 0.999). However, the EUS-CDS group demonstrated higher rates of late AEs (33.33% vs. 5.88%, *p* = 0.088) and LAMS dysfunction (16.67% vs. 5.88%, *p* = 0.603) compared to the EUS-GBD group [51].

## 4. EUS-Guided Gastroenterostomy (EUS-GE)

### 4.1. The Procedure

**Figure 3:**
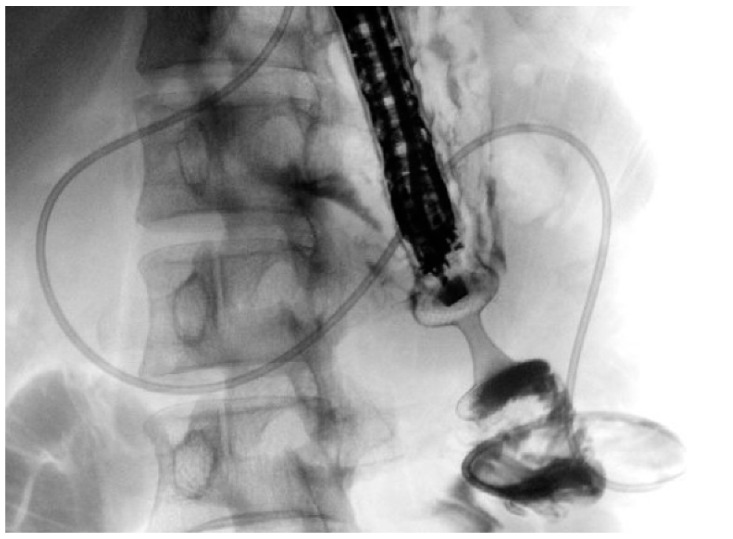
Endoscopic ultrasound-guided gastroenterostomy (EUS-GE). Fluoroscopic view of EUS-GE with contrast medium flow through the Lumen-Apposing Metal Stent (LAMS) lumen from the jejunum to the stomach. The copyright of the image belongs to the authors.

Historically, the treatment options for GOO have included surgical gastroenterostomy (S-GE) and enteral stenting (ES), which involves placing a metal stent through the stenosis [52]. While S-GE is highly effective, it is a surgical intervention associated with a notable risk of morbidity due to surgical complications and is often not feasible for patients unfit for surgery, such as those with advanced oncological disease. ES, on the other hand, is a minimally invasive procedure with a lower risk of AEs and good short-term efficacy [53]. However, it is frequently associated with stent obstruction, necessitating reintervention [54,55]. In this context, EUS-guided gastroenterostomy (EUS-GE) has emerged as a minimally invasive endoscopic alternative to S-GE for managing GOO. Like S-GE, EUS-GE involves creating a gastro-jejunal anastomosis, approximately 20 mm in size, by placing a fully covered dumbbell-shaped Lumen-Apposing Metal Stent (LAMS) under EUS guidance downstream of the obstruction (Figure 3) [13,56,57].

### 4.2. The Technique

Three main techniques for performing EUS-GE have been described in the ESGE technical review [9]. The choice of technique depends on the endoscopist’s expertise, technical background, available equipment, and patient-specific factors. Across all EUS-GE techniques, the most challenging step is the precise identification and stabilization of the target jejunal loop, followed by the deployment of the LAMS. The first jejunal loop just above the Treitz angle is often preferred due to the stability provided by the Treitz ligaments and its proximity to the stomach, avoiding more distal, and consequently more mobile, loops.

Techniques for EUS-GE were as follows:

(a) Direct Technique: This approach involves identifying the target loop under EUS guidance and puncturing it with a 19-gauge FNB needle to fill it with saline, often combined with dye and contrast medium, to confirm positioning under fluoroscopy. The LAMS is then deployed over a guidewire, creating the anastomosis [9];
(b) Device-Assisted Technique: Additional support devices such as dilation/stone extraction balloons or double-balloon enteroscopes (DBEs) are used. After passing a guidewire through the stenosis under fluoroscopy, the balloon catheter is advanced over the wire. The target loop is identified with EUS, and the balloon is inflated with saline and the contrast medium to stabilize it. The subsequent steps mirror the direct technique: puncture with a 19-gauge FNB needle, loop filling, and LAMS deployment over the wire [9];
(c) Wireless Endoscopic Simplified Technique (WEST): First described by Bronswijk M et al. in 2020, this technique involves placing a naso-enteric tube in the jejunal loop downstream of the stenosis [58]. The jejunal loop is filled with saline and dye (e.g., indigo carmine or methylene blue) under fluoroscopic guidance to prevent diffuse distension of other bowel segments, which could complicate identification. The target loop is visualized under EUS by observing the liquid flow and the naso-enteric tube’s presence. Antispasmodic agents like Scopolamine-N-butyl bromide may be administered during this step. LAMS deployment is performed in a single step, potentially reducing procedure time and minimizing the risk of AEs associated with device exchanges [9,58].

#### 4.2.1. Novel Approaches

Chen Y-I and colleagues recently introduced the use of a through-the-scope exchangeable dual-balloon enteroclysis catheter (DUBX Naja, Chess Medical, Montreal, QC, Canada) for stabilizing the target jejunal loop during EUS-GE. This innovative device improves procedural safety and reduces the risk of complications by providing enhanced stability and precision during the procedure [59].

#### 4.2.2. Comparison of Techniques

The superiority of one EUS-GE technique over another remains a subject of debate. A multicenter retrospective study by Monino L et al. compared the WEST with the over-the-wire technique in tertiary centers [60]. The WEST technique showed a significantly higher TS rate (95.1% vs. 73.3%; eRR 3.2, 95% CI 0.94–10.9; *p* = 0.01). AEs, particularly stent misdeployment, were more frequent in the over-the-wire group compared to the WEST group (46.7% vs. 14.6%; eRR 2.3, 95% CI 1.2–4.5; *p* = 0.007). Major AEs were also higher in the over-the-wire group (13.3% vs. 7.3%), although this difference was not statistically significant (*p* = 0.22). Mortality rates between the two techniques were comparable (6.7% vs. 2.4%; *p* = 0.57) [60].

### 4.3. Indications

According to the ESGE guidelines, the primary indication for EUS-GE is malignant GOO as an alternative to ES or S-GE. EUS-GE should be performed exclusively in expert settings within tertiary referral centers following multidisciplinary discussion [13]. In Western countries, the most common cause of mGOO is pancreatic ductal adenocarcinoma, with other etiologies including gastric cancer, primary or metastatic malignancies of the duodenum and ampulla, advanced gallbladder carcinoma or cholangiocarcinoma, gastric carcinoids, gastrointestinal stromal tumors (GISTs), and gastric leiomyosarcomas [61]. The increasing role of neoadjuvant treatments, particularly for PDAC, has significantly improved survival rates for these patients [62]. In this context, EUS-GE, like EUS-BD, is transitioning from being a palliative option for unresectable cases to a feasible procedure in potentially resectable patients, with growing evidence supporting its use as a bridge-to-surgery intervention [44,63,64]. GOO can also result from benign conditions, such as chronic pancreatitis, peptic ulcer disease, and large antral polyps [20,21]. While long-term data are still lacking, EUS-GE has demonstrated its role in the benign setting, both as a definitive treatment and as a bridge to surgery [65,66].

### 4.4. Controindications

Absolute contraindications for EUS-GE include uncontrolled coagulopathy, malignant or refractory ascites, and peritoneal carcinomatosis [9,13]. Massive and refractory ascites indicate a significant tumor burden, which increases the risk of downstream enteric obstructions and infection if an electrocautery catheter is used. However, mild ascites is not considered an absolute contraindication. In such cases, prophylactic antibiotics are recommended, and the placement of abdominal drainage may further enhance procedural safety. Diffuse gastric wall infiltration is also an absolute contraindication, as it may impair gastric motility and increase the thickness of the gastric wall. This necessitates a higher cutting current, which raises the risk of bleeding and significantly increases the likelihood of LAMS misdeployment [67].

### 4.5. Efficacy

EUS-GE is recommended in a high-expertise setting as an alternative to ES or S-GE for the palliative treatment of mGOO [13]. Evidence on EUS-GE has evolved through various stages, starting with a retrospective series comparing it to ES and S-GE, followed by prospective observational studies, and culminating in the recently published RCT by Teoh AYB et al., which directly compared EUS-GE with ES [68].

Vanella G et al. conducted a prospective cohort study evaluating the outcomes of 70 patients with malignant GOO treated by EUS-GE [69]. The majority of the patients had pancreatic cancer (75.7%), with 60.0% presenting metastatic disease. The EUS-GE procedures were predominantly performed using the Wireless Endoscopic Simplified Technique (97.1%) and employed a 20 × 10 mm ec-LAMS in 97.1% of cases. The study’s primary outcomes were TS, defined as successful LAMS placement between the stomach and jejunum; CS, defined as achieving a Gastric Outlet Obstruction Scoring System (GOOSS) score of 2 (soft solids) post-procedure; and AEs. Secondary outcomes included symptom recurrence, time to resumption of fluid and solid oral intake, hospital stay, time to chemotherapy resumption, and overall survival. Both TS and CS rates were 97.1%, with a median time to achieving CS of 1.5 days (IQR 1–2). The overall AE rate was 12.9%, with moderate bleeding being the most frequent complication (5.7%), managed with endoscopic epinephrine injection. Two fatal AEs occurred, both involving post-procedural severe cholangitis related to suboptimal biliary drainage before EUS-GE. After a median follow-up of 105 days (IQR 49–187), 61.4% of patients resumed chemotherapy (CT) with a median time-to-CT of 19 days (IQR 14–26). Symptom recurrence was observed in 7.6% of patients, occurring after a median of 78 days (IQR 28–164). Kaplan–Meier analysis revealed an estimated mean symptom-free survival (SFS) of 480 days (95% CI 426–534), with SFS probabilities of 96.7%, 90.6%, and 85% at 3, 6, and 12 months, respectively. During the same study period, 35 patients with mGOO underwent duodenal stent placement, primarily due to contraindications for EUS-GE. A 1:1 matched subgroup analysis (matched for ASA score and presence of peritoneal carcinomatosis) compared 28 patients treated with EUS-GE to 28 patients treated with duodenal stenting. The results showed that EUS-GE was associated with a significantly higher CS rate (100% vs. 75.0%, *p* = 0.006) and significantly lower symptom recurrence rates (3.7% vs. 33.3%, *p* = 0.02) compared to duodenal stenting [69].

The previously mentioned international multicenter RCT involving seven high-volume centers was the first to compare EUS-GE and ES as palliative treatments for mGOO in patients with unresectable primary gastro-duodenal or pancreaticobiliary malignancy. Data from 97 patients were analyzed, who were randomly assigned to EUS-GE (48 patients) or ES (49 patients). The primary outcome was the 6-month reintervention rate, which is defined as the need for additional procedures to address stent dysfunction. Secondary outcomes included TS (correct stent placement), CS (an improvement of 1 point in the GOOS within 3 days post-procedure), AEs within 30 days, 30-day post-procedure mortality, stent patency duration, GOOS at one month, and quality of life. All procedures were performed by experienced endoscopists; EUS-GE was conducted using the EPASS technique [70]. The results showed that EUS-GE had a lower 6-month reintervention rate compared to ES, with two (4%) patients in the EUS-GE group versus 14 (29%) patients in the duodenal stent group [*p* = 0.0020; RR 0.15 (95% CI 0.04–0.61)]. Stent patency was also longer in the EUS-GE group [median stent patency not reached in either group; HR 0.13 (95% CI 0.08–0.22), *p* < 0.0001]. The two patients in the EUS-GE group who required reintervention underwent ES placement. Subsequently, both experienced tumor ingrowth, which was managed by placing an additional ES. In the ES group, among patients requiring reintervention, 12 had tumor ingrowth, necessitating the placement of an additional ES. One patient experienced stent obstruction caused by food, which was cleared endoscopically, and another had proximal stent migration into the stomach, requiring ES removal and placement of a new ES. There was no statistically significant difference in TS and CS between the two groups: 46 (96%) out of 48 in the EUS-GE group versus 49 (100%) out of 49 in the duodenal stent group [RR 0.96 (95% CI 0.90–1.02); *p* = 0.242] and 48 (100%) versus 45 (92%) [RR 1.09 (95% CI 1.00–1.18) *p* = 0.117], respectively. In the EUS-GE group, the two patients who experienced technical failure, as previously mentioned, received an ES; there were no cases of stent misdeployment. In the ES group, four patients experienced technical failure; all of them underwent an additional procedure involving the placement of another ES.

Van Tran K et al. recently published a comprehensive network meta-analysis (NMA) evaluating four primary treatment modalities for managing malignant GOO: S-GE, stomach-partitioning gastrojejunostomy (PGJ), ES, and EUS-GE [57]. The analysis included data from 40 studies with a total of 3716 patients. The primary outcome was clinical efficacy, measured by the reintervention rate, while secondary outcomes included CS, complication rates, 30-day mortality, and length of hospital stay (LOS). To assess combined safety and efficacy, the reintervention rate was also analyzed alongside 30-day mortality and complication rates. The overall outcomes reported were CS at 88.9%, AEs at 20.7%, 30-day mortality at 5.4%, and reintervention at 13.9%. Compared to ES, the risk of reintervention was significantly lower for PGJ, EUS-GE, and S-GE, with relative risks of 0.14, 0.27, and 0.51, respectively. Among these, PGJ and EUS-GE demonstrated the highest probabilities of reducing reintervention rates (P scores: 0.90 and 0.72), while ES had the lowest (P score: 0.00). For preventing reinterventions due to obstruction, PGJ and EUS-GE emerged as the most effective treatments, with P scores of 0.88 and 0.73, respectively. PGJ also ranked highest in preventing reinterventions due to AEs, achieving a P score of 0.81. In terms of clinical success, PGJ showed a higher probability compared to ES (RR 1.22), while EUS-GE and S-GE exhibited similar success rates to ES but with lower certainty. PGJ had the highest clinical success probability (P score: 0.95), followed by EUS-GE (P score: 0.69). ES and S-GE ranked lowest in this metric, with P scores of 0.20 and 0.17, respectively. Regarding LOS, ES and EUS-GE demonstrated similar durations, while S-GE and PGJ were associated with longer hospital stays. ES had the highest probability of minimizing LOS (P score: 0.92), followed by EUS-GE (P score: 0.75). Conversely, S-GE and PGJ showed the lowest probabilities (P scores: 0.28 and 0.05, respectively) [57].

These findings align with those reported in a meta-analysis by Miller C et al., which analyzed data from 16 studies involving a total of 1541 patients undergoing palliative treatment for malignant GOO using EUS-GE, S-GE, and ES [54]. The primary outcomes included CS without recurrent GOO and AEs, while the secondary outcomes were TS and length of hospital stay (LOS). EUS-GE demonstrated a significantly higher rate of CS without recurrent GOO compared to ES or S-GE combined (OR: 2.60; 95% CI: 1.58–4.28). Subgroup analysis further revealed that EUS-GE had a notably higher CS rate compared to ES alone (OR: 5.08; 95% CI: 3.42–7.55). However, no statistically significant difference was observed when EUS-GE was compared to S-GE alone (OR: 1.94; 95% CI: 0.97–3.88). In terms of technical success, EUS-GE was associated with significantly lower rates compared to both ES or S-GE combined (OR: 0.32; 95% CI: 0.16–0.64) and S-GE alone (OR: 0.17; 95% CI: 0.06–0.49). However, no significant difference in TS was observed between EUS-GE and ES alone (OR: 0.44; 95% CI: 0.18–1.12). Regarding hospital stay lengths, no significant differences were identified between EUS-GE and either ES or S-GE combined or when compared to ES or S-GE alone [54].

### 4.6. Safety

EUS-GE is not without AEs, with the most common intraprocedural complications including stent misdeployment, perforation, leakage leading to peritonitis, bleeding, stent misplacement, migration, or dislodgement [67]. Long-term AEs frequently involve abdominal pain, which is the most common, and erosion or ulceration of the opposing gastric or jejunal wall caused by the LAMS mesh [69].

In the recent RCT by Teoh AYB et al. comparing EUS-GE to ES, no statistically significant difference was observed in 30-day mortality between the two groups [68]. In the EUS-GE group, 10 patients (21%) died within 30 days, compared to 6 patients (12%) in the ES group (*p* = 0.286; RR 1.70 [95% CI 0.67–4.32]). Causes of death in the EUS-GE group included disease progression (3 cases), stroke (2), pleural effusion (1), pulmonary embolism (1), cholangitis (1), obstructive jaundice (1), and pneumonia (1). In the ES group, causes of 30-day mortality included disease progression (2 cases), cardiac arrest (1), pulmonary embolism (1), cholangitis (1), and recurrent hypoglycemia (1). Similarly, there was no significant difference in 30-day AEs, with 11 (23%) in the EUS-GE group and 12 (24%) in the ES group (*p* = 1.00; RR 0.94 [95% CI 0.46–1.92]) [68].

A meta-analysis assessing AEs associated with EUS-GE across 36 studies reported pooled TS and CS rates of 96.9% (95% CI 95.9–98.0) and 90.6% (95% CI 88.5–92.7), respectively [71]. The pooled AE rate was 13.0% (95% CI 10.3–15.7), with a significantly lower rate of AEs observed with the WEST (8.4%) compared to other techniques (17.3%) [58]. Serious AEs were rare, occurring in 1.2% (95% CI 0.7–1.8) of cases, with no significant difference between free-hand and other techniques. Procedure-related mortality was extremely low, at 0.3% (95% CI 0.0–0.7) [71]. Misdeployment, the most frequent AE (4.6%, 95% CI 3.2–6.0), was less common with the free-hand technique (2.8%) compared to mixed techniques (7.1%). Other complications, such as perforation, peritonitis without perforation, and bleeding, had very low incidences (≤0.4%, 95% CI 0.0–0.9). Delayed stent migration and occlusion were rare, with pooled incidences of 0.5% (95% CI 0.0–1.1) and 0.8% (95% CI 0.2–1.3), respectively [71].

Ghandour B et al. published a retrospective multicenter study proposing a classification system for LAMS misdeployment, identifying four distinct types [67]:

- Type 1: Deployment of the distal flange in the peritoneum and the proximal flange in the stomach without evidence of enterotomy. This was the most common type, occurring in 63.1% of cases. In this scenario, LAMS removal and defect closure with an over-the-scope clip is the most common rescue strategy;
- Type 2: Deployment of the distal flange in the peritoneum and the proximal flange in the stomach, with evidence of enterotomy confirmed by endoscopy or fluoroscopy. This type was reported in 30.4% of cases. In this context, after the removal of the first LAMS, the procedure can be repeated with the placement of a new LAMS, utilizing the existing oro-jejunal tube as a target and continuing to distend it [72]. Alternatively, the LAMS-in-LAMS bridging technique could serve as a viable option in the hands of experienced operators [73,74,75];
- Type 3: Deployment of the distal flange in the small bowel and the proximal flange in the peritoneum. This type was less frequent, accounting for 2.2% of cases, and its management is preferably surgical;
- Type 4: Deployment of the proximal flange in the stomach and the distal flange in the colon, observed in 4.3% of cases. In this setting, one option should be to wait for at least 48 h for gastro-colonic tract maturation, then proceed to LAMS removal and to both defects closure with over-the-scope clips. Alternatively, surgery should be an option.

Results from the previously mentioned NMA indicated that EUS-GE had a lower risk of complications compared to ES (RR 0.58), while PGJ had a comparable risk (RR 1.43) [57]. EUS-GE was found to have the highest probability of being the safest treatment option (P score: 0.99), followed by ES (P score: 0.62). Regarding 30-day mortality, EUS-GE and PGJ had similar safety profiles (P scores: 0.82 and 0.79), with ES and SGE having lower probabilities (P scores: 0.21 and 0.18) [57].

Consistent with these findings, another meta-analysis reported that EUS-GE was associated with significantly fewer AEs compared to ES or SGJ combined (OR: 0.34; 95% CI 0.20–0.58) [54]. While subgroup analysis revealed no significant differences in AE rates between EUS-GE and ES alone (OR: 0.57; 95% CI 0.29–1.14), EUS-GE demonstrated significantly fewer AEs compared to SGJ alone (OR: 0.17; 95% CI 0.10–0.30). Despite the generally favorable safety profile, concerns about potential complications, particularly stent misdeployment, persist.

## 5. Endoscopic Treatment of Double Obstruction

Concomitant malignant DBO and GOO still represent a challenging clinical condition to treat. The last years have seen the development of T-EUS procedures, firstly with EUS-BD interventions, which now have an established role recognized by international societies in case of ERCP failure, and secondly with the diffusion of EUS-GE as the first treatment choice for GOO, especially in tertiary referral centers [13,68]. There is no strong evidence on this specific challenging topic, but the scientific community is growing interested in producing high-quality data. Current evidence on this specific topic is limited by several factors. Firstly, most studies are retrospective and lack controlled designs. Secondly, most investigations into EUS-BD procedures include a variety of approaches (EUS-HGS, EUS-CDS, EUS-GBD, and EUS-AS) performed with different techniques and devices (SEMS, LAMS) without conducting subgroup analyses for each specific procedure. Thirdly, the management of GOO has evolved from ES to EUS-GE, leading to the inclusion of mixed cases treated with both methods in many studies. Lastly, most available studies fail to classify patients based on the type of duodenal stenosis and oncological status, further limiting the interpretability and applicability of the data. In this section of our review, we aimed to summarize the results from more relevant studies on this topic, focusing on the impact of T-EUS procedures in this setting (Table 1).

### Evidence

In 2016, Ogura T et al. published a single-center retrospective study including 39 patients affected by mDBO and GOO, comparing stent patency and safety of EUS-CDS (13 patients) and EUS-HGS (21 patients) in this setting [76]. All patients underwent ES for GOO treatment 1 week before the EUS-BD procedure. Most of the patients were affected by pancreaticobiliary tumors, without any differences between the two groups concerning baseline characteristics and the type of duodenal stenosis, according to Mutignani M et al.’s classification [83]. All EUS-CDS were performed using the over-the-wire technique and FC-SEMS. The patency of both ES and SEMS used for the EUS-BD procedures was calculated from the stent placement to stent dysfunction (stent obstruction, migration, cholangitis), patient death, or last follow-up. The authors performed a Kaplan–Meier analysis comparing duodenal and biliary stent patency and overall survival (OS) according to the type of EUS-BD. According to ES patency, no significant difference was observed between the EUS-CDS and the EUS-HGS group (EUS-CDS median 42 days, EUS-HGS median 113 days; HR 0.557, 95% CI 0.335–1.467; *p* = 0.343). Instead, biliary stent patency was significantly longer in the EUS-HGS group than in the EUS-CDS group (EUS-CDS median 43 days, EUS-HGS median 133 days; HR 0.492, 95% CI 0.239–1.013; *p* = 0.0497). As for the ES patency, also for overall survival, no significant differences were observed between the two groups (EUS-CDS median 98 days, EUS-HGS median 133 days; HR 0.648, 95% CI 0.309–1.359; *p* = 0.247). Furthermore, the authors performed always KM analysis for OS, ES, and biliary stent patency adjusted for six variables selected as covariates (age, pancreaticobiliary carcinoma, type of duodenal stenosis, length of duodenal stenosis, size of the tumor). From this analysis, ES and biliary stent patency were significantly longer in the EUS-HGS group than in EUS-CDS group (ES patency: EUS-CDS median 34 days, EUS-HGS median 113 days; HR 0.415, 95% CI 0.175–0.984; *p* = 0.046; biliary stent patency: EUS-CDS median 37 days, EUS-HGS median 133 days; HR 0.391, 95% CI 0.156–0.981; *p* = 0.045). Concerning AEs, on logistic regression analysis, EUS-CDS was associated with reflux cholangitis (OR 10.285, 95% CI 1.686–62.733; *p* = 0.012) [76].

Two years later, Hamada T et al. published an international multicenter retrospective cohort study including 110 patients with dMBO and GOO, mostly affected by unresectable pancreatic cancer [77]. Authors excluded patients undergoing previous S-GE, PTBD, surgical biliary bypass, or patients with surgically altered anatomy. They were divided into three groups based on the occurrence of biliary and duodenal obstruction: group 1 (n = 67), dMBO before GOO; group 2 (n = 29), dMBO simultaneous with GOO and group 3 (n = 14), dMBO after GOO. Patients were also classified according to the type of duodenal stenosis: 45, 46, and 19 patients with type I, type II, and type III obstructions, respectively. Also, in this study, ES represented GOO treatment. Concerning biliary drainage, ERCP, EUS-CDS, and EUS-HGS were performed in 90 (82%), 10 (9.1%), and 10. (9.1%) patients. The median time to recurrent biliary obstruction (TRBO) for all cases, which was the primary outcome of this study, was 450 days (95% CI: 212–666 days). ERCP, EUS-CDS, and EUS-HGS showed a TRBO of 455, 344, and 137 days, respectively. A total of 37 patients (34%) had recurrent biliary obstructions; 26 (39%), 9 (31%), 2 (14%) in group 1, group 2, and group 3, respectively (*p* = 0.21); 19 (42%), 13 (28%), 5 (26%) in type I, type II, and type III duodenal stenosis groups, respectively (*p* = 0.28). A total of 15 (14%) AEs were observed: 8 (8.9%) AEs in the ERCP group, 2 (20%) in the EUS-CDS group, and 5 (50%) in the EUS-HGS group (*p* = 0.0029) [77].

In 2021, Debourdeau A et al., in their retrospective single-center study, evaluated the feasibility of performing same-session duodenal–biliary stenting in patients with concomitant biliary and duodenal obstruction and the impact of EUS-HGS in the setting of double malignant obstruction [78]. Hospital stays and bilio-duodenal reintervention rates represented the primary and secondary endpoints. Statistical analysis was performed comparing outcomes of patients in same-session (n = 16) or two-session groups (n = 15) and in EUS-HGS (n = 11) and duodenal route (ERCP, n = 8; PTBD, n = 11; EUS-CDS, n = 1) groups. According to the results, the same-session group was associated with a significantly shorter hospital stay (7.5 vs. 12.6 days; *p* = 0.04) compared to two-session groups. The EUS-HGS group, compared to the trans-duodenal BD route group, showed a lower AE rate (55% vs. 18.2%, *p* = 0.07) and a lower biliary reintervention rate (30% vs. 9.1%, *p* = 0.37) [78].

In 2021, Mangiavillano B et al. published the first study evaluating the role of EUS-BD with LAMS (EUS-CDS and EUS-GBD via the duodenal route) through the mesh of a previously placed ES in 23 patients with double malignant obstruction, most of whom had locally advanced pancreatic cancer [79]. In most cases, EUS-BD was the first-line treatment (17/23, 73.9%), and in 19/23 patients (82.6%), the EUS-BD procedures were performed after a median of 7 days (IQR 5–15; range 2–127 days) following ES placement. EUS-GBD and EUS-CDS were performed in 14/23 (60.9%) and 9/23 (39.1%) cases, respectively. The technical failure occurred in only one case (4.4%) of EUS-CDS, where there was a misdeployment of the distal flange. This was followed by LAMS removal and conversion to EUS-GBD without significant clinical consequences. All patients resumed oral feeding the day after ES placement, and a substantial decrease in serum bilirubin levels was observed following the EUS-BD procedures. No patients experienced jaundice recurrence during a median survival of 241 days (IQR 81–387), and no adverse events related to ES or LAMS were reported [79].

The feasibility of performing single-session EUS-HGS and EUS-GE in patients with double obstruction was evaluated by Canakis A et al. in their retrospective, two-center study, which included 23 patients [80]. In 15 of the 23 patients (65%), ERCP was unsuccessful due to various reasons, including duodenal stenosis, failed cannulation, ampullary region infiltration, and surgically altered anatomy. Technical success was achieved in 100% of cases in the EUS-HGS group and in 95.6% (22/23) of cases in the EUS-GE group; in the latter, one patient experienced LAMS misdeployment, which was managed by placing a through-the-LAMS fully covered SEMS, with no clinical impact. Regarding clinical efficacy, all patients were able to resume at least a soft-solid diet indefinitely. A 50% reduction in total serum bilirubin was achieved in 72.7% (16/22) of patients, though the authors did not report data on reasons for failure or rescue management. After a median follow-up of 178.4 ± 185.9 days, jaundice recurrence occurred in 3 of 22 patients (14%), requiring biliary reinterventions. One patient underwent PTBD for the right biliary system, another had a SEMS-in-SEMS placement for EUS-HGS SEMS occlusion with a slight bilirubin reduction despite massive metastatic liver disease, and the third patient required an EUS-guided stent exchange for a benign biliary stricture in the context of chronic pancreatitis complicated by duodenal stenosis and biliary obstruction. A total of 5 AEs (21.7%) were recorded: two mild and three moderate (EUS-HGS SEMS dislodgment, hepatic abscess, and biloma) [80].

Vanella G et al. conducted a retrospective analysis of 93 patients (103 procedural combinations) to compare outcomes of different endoscopic strategies for managing double obstruction (CABRIOLET study) [23]. The study’s primary aim was to assess dysfunction, defined as recurrence of jaundice or GOO, in terms of rate (proportion) and time-to-event (dysfunction-free survival, DFS). The study included only patients who underwent biliary and GOO procedures within a 180-day interval and had a minimum follow-up of 30 days. Most patients had pancreatic cancer (73%) and were metastatic at diagnosis (57%). The procedural combinations were as follows: ES+transpapillary SEMS (TPS) in 38 patients, ES+EUS-CDS in 10 patients, ES+EUS-HGS in one patient, EUS-GE+TPS in 29 patients, EUS-GE+EUS-CDS in 19 patients, and EUS-GE+EUS-HGS in 6 patients. The ES+EUS-CDS group had a significantly higher rate of primary failure (40%, *p* = 0.02) and dysfunction (83%, *p* = 0.002) compared to the other groups (ES+TPS 53%, EUS-GE+EUS-CDS 31%, EUS-GE+TPS 18%, EUS-GE+EUS-HGS 0). Kaplan–Meier analysis revealed that the 6-month DFS probability for the ES+EUS-CDS group (22.2%) was significantly lower compared to other groups (ES+TPS 46.7%, EUS-GE+EUS-CDS 41%, EUS-GE+TPS 72.2%, EUS-GE+EUS-HGS 100%; log-rank *p* = 0.004). When focusing on biliary dysfunction, excluding GOO recurrence, the ES+EUS-CDS group also showed a higher rate (50%), although this was not statistically significant (*p* = 0.2). To identify predictors of dysfunction, the authors performed a univariate analysis, which showed that type III duodenal stenosis significantly increased the probability of dysfunction (HR 2.6, [1.2–5.4]). Further analysis revealed that specific combinations, such as ES+TPS and ES+EUS-CDS, had a higher dysfunction risk compared to EUS-GE+EUS-HGS (HR 2.3 [1.1–5.2] and 6.5 [2.2–19.2], respectively). In multivariate analysis, both type III duodenal stenosis (HR 3.2 [1.5–6.9]) and the ES+EUS-CDS combination (HR 5.6 [2.1–15.7]) were identified as independent predictors of dysfunction [23].

An international multicenter retrospective study by Bronswijk M et al. recently compared outcomes of same-session double EUS-guided bypass (EUS-GE + EUS-BD) with S-GE and hepaticojejunostomy (S-HJ) in 154 patients, 53 in the EUS group and 101 in the surgery group [81]. In the EUS-BD cohort, EUS-CDS was performed in 29 patients, EUS-HGS in 16, EUS-AS in 7, and EUS-RVS in 1. The study’s primary endpoint was clinical success (CS), defined as a GOOS ≥2, along with a post-procedural decrease in bilirubin >50% within one month or successful treatment of cholangitis. Baseline comparisons showed that patients in the EUS group had higher ASA scores and Charlson Comorbidity Index scores (9.0 [IQR, 7.0–10.0] vs. 7.0 [IQR, 5.0–9.0], *p* < 0.001) compared to those in the surgery group. Technical success (TS) rates were similar between the groups (96.2% vs. 100%, *p* = 0.117), as were CS rates (90.6% vs. 82.2%, *p* = 0.234). However, per-protocol analysis indicated a significantly higher CS rate in the EUS group (94.1% vs. 82.2%, *p* = 0.049), mainly due to a higher clinical failure rate in the S-GE group. The EUS-GE group also had a significantly shorter median time to oral intake (0 days [IQR, 0–1] vs. 6 days [IQR, 3–7], *p* < 0.001). For different EUS-BD approaches, no differences were observed in TS and CS rates when comparing EUS-HGS with other approaches. The surgery group, however, had a significantly higher overall adverse event (AE) rate (11.3% vs. 34.7%, *p* = 0.002) and severe AE rate (3.8% vs. 19.8%, *p* = 0.007) compared to the EUS group. Among EUS-BD procedures, EUS-AS had a significantly higher AE rate compared to EUS-HGS (0% vs. 42.9%, *p* = 0.020). Secondary outcomes further favored the EUS group, showing a significantly shorter hospital stay (4.0 days [IQR, 3–9] vs. 13 days [IQR, 9–22], *p* = 0.001) and shorter procedural time (51 min [IQR, 32–77] vs. 198 min [IQR, 139–263], *p* < 0.001). A sub-analysis revealed that EUS-CDS was associated with a shorter hospital stay (4 days [3–9] vs. 4 days [3–9], *p* = 0.026) and procedural time (50 min [IQR, 32–75] vs. 47 min [IQR, 32–73], *p* = 0.014) compared to EUS-HGS. No differences in biliary obstruction recurrence were observed between the EUS and surgery groups or among the various EUS-BD approaches during follow-up [81].

Fugazza A et al. recently published the B-GOOD study, a retrospective multicenter analysis comparing outcomes of EUS-GE and ES in 77 patients with malignant double obstruction who had previously undergone EUS-CDS [82]. The primary outcomes were the overall AE rate and biliary and GOO dysfunction; secondary outcomes included TS, CS, procedural time, and hospital stay. EUS-GE and ES had similar AE rates (8% vs. 17.3%, *p* = 0.27), with one case of stent migration and one case of post-procedural bleeding in the EUS-GE group, and three cases of bleeding, one case of ES obstruction, and five cases of GOO symptom recurrence in the ES group. Additionally, no significant differences were found between the EUS-GE and ES groups in terms of EUS-CDS dysfunction (12.5% vs. 17.3%; *p* = 0.74) and reintervention rate (12.5% vs. 23.1%; *p* = 0.36). Both groups also showed similar results regarding TS (96% vs. 100%, *p* = 0.32) and CS rates (100% vs. 98%, *p* = 1.0), as well as hospital stay (3 ± 2 vs. 5 ± 2 days; *p* = 0.15) and procedural time (30.0 ± 13.6 vs. 25.0 ± 12.1 min; *p* = 0.20) [82].

New data highlighting the apparent superiority of EUS-HGS over EUS-CDS in malignant double obstruction comes from the prospective comparative study by Vanella G et al. (CABRIOLET_Pro study), which included 20 patients (75% with pancreatic cancer, 50% with metastatic disease) [24]. All patients underwent EUS-GE for GOO management, with seven receiving EUS-CDS and 13 receiving EUS-HGS for biliary drainage. Technical success (TS) rates were similar between EUS-CDS and EUS-HGS (100% vs. 100%, *p* = 1), as were clinical success (CS) [defined as achieving a >50% reduction in bilirubin levels within 14 days after stent placement for BO,11 and a post-procedural GOOSS ≥2 (ability to eat at least soft solids) within 14 days after stent placement for GOO] rates (100% vs. 92.3%, *p* = 0.5). However, EUS-CDS was associated with a higher rate of severe post-procedural adverse events (42.9% vs. 7.7%, *p* = 0.067), mainly due to severe and fatal acute cholangitis, as well as a significantly higher rate of biliary dysfunction (71.4% vs. 16.7%, *p* = 0.02, relative risk = 4.3 [1.1–16.5], Number Needed to Harm = 1.8) during follow-up. All five cases of dysfunction in the EUS-CDS group were attributed to ascending cholangitis caused by food impaction in the LAMS (Type 5 according to the L.A.M.S. Classification by *Vanella G* et al.) [28,29]. In the EUS-HGS group, the two dysfunction cases were managed with balloon swipes, EUS-AS, and antibiotic therapy. Kaplan–Meier analysis demonstrated a significantly shorter dysfunction-free survival for EUS-CDS compared to EUS-HGS (39 [15–62] vs. 268 [192–344] days, log-rank *p* = 0.0023, hazard ratio = 7.8 [1.4–44.2]), with a dysfunction-free survival probability of 53% vs. 100%, respectively [24].

## 6. Clinical Scenario

**Figure 4:**
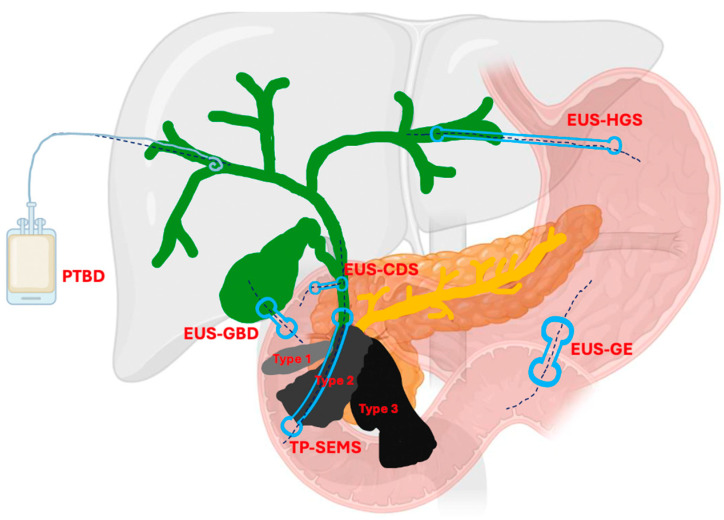
Management of malignant double obstruction (MBO). PTBD, percutaneous transhepatic biliary drainage. EUS-GBD, endoscopic ultrasound-guided gallbladder drainage. EUS-CDS, Endoscopic Ultrasound-Guided Choledochoduodenostomy. TP-SEMS, Transpapillary Self-Expandable Metal Stent. EUS-GE, Endoscopic Ultrasound Gastroenterostomy. EUS-HGS, Endoscopic Ultrasound-Guided Hepaticogastrostomy. Type 1, type 2, and type 3 duodenal stenosis according to Mutignani M et al. classification [83]. The copyright of the image belongs to the authors.

**Figure 5:**
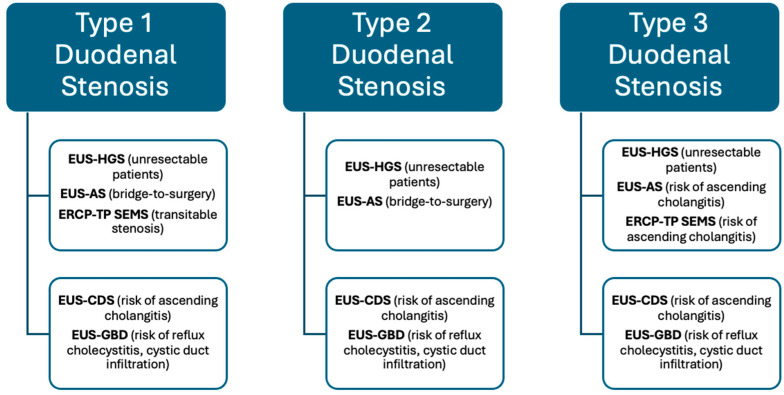
Biliary drainage strategies according to duodenal stenosis type and oncological status of the patient, considering EUS-GE the main treatment for gastric outlet obstruction. EUS-HGS, Endoscopic Ultrasound-Guided Hepaticogastrostomy. EUS-AS, Endoscopic Ultrasound-Guided Antegrade Stenting. ERCP-TP-SEMS, Endoscopic Retrograde Cholangiopancreatography Transpapillary Self-Expandable Metal Stent. EUS-CDS, Endoscopic Ultrasound-Guided Choledochoduodenostomy. EUS-GBD, endoscopic ultrasound-guided gallbladder drainage. The copyright of the image belongs to the authors.

The choice of endoscopic technique for managing malignant double obstruction (DO) depends not only on the location of the MBO, whether distal or proximal and the site of duodenal obstruction based on the Mutignani M et al. classification proposed in 2007 but also on the oncological staging of the patient [83]. The authors of this study described three types of duodenal stenosis: type 1, where the obstruction is at the level of the duodenal bulb or upper duodenal genu without papillary involvement; type 2, where the obstruction involves the second part of the duodenum, including the major papilla; and type 3, where the stenosis affects the third part of the duodenum, leaving the ampullary region unaffected and accessible [83]. As outlined in the dedicated section, growing evidence supports EUS-GE as the primary treatment for GOO, particularly in tertiary referral centers, due to its high clinical efficacy in both the short and long term and its favorable safety profile [68]. From this perspective, in cases of DO, the preferred treatment of GOO should be EUS-GE, provided there are no technical or clinical contraindications [9]. The primary consideration would then focus on selecting the ideal route for biliary drainage. In this section of our review, we aim to present various clinical scenarios with corresponding endoscopic management alternatives, classifying patients based on the combination of the site of BO, the type of GOO, and their oncological staging (Figure 4 and Figure 5).

### 6.1. Distal Malignant Biliary Obstruction and Type I Gastric Outlet Obstruction

In patients with dMBO and type I GOO, the papilla is theoretically unreachable but unaffected by stenosis. In this scenario, the initial approach could involve gently attempting to pass the stenosis with the duodenoscope, guided both endoscopically and fluoroscopically, to reach the second portion of the duodenum and perform ERCP with transpapillary stenting. If this option is not feasible, another solution could be to place a short ES across the stenosis and, after waiting at least 24–48 h for it to expand fully, attempt to reach the ampullary area by passing the scope through the ES. This solution has two main limitations: first, the need for two separate procedures, which prolongs hospital stay, increases symptom burden, and requires two rounds of sedation; and second, the patient would undergo two endoscopic procedures, EUS-GE and ES, both aimed at the same goal. This would result in a higher risk of AEs despite both procedures having a good safety profile. Obviously, in this scenario, EUS-CDS and EUS-GBD could offer viable solutions, but certain considerations should be made.

**Figure 6:**
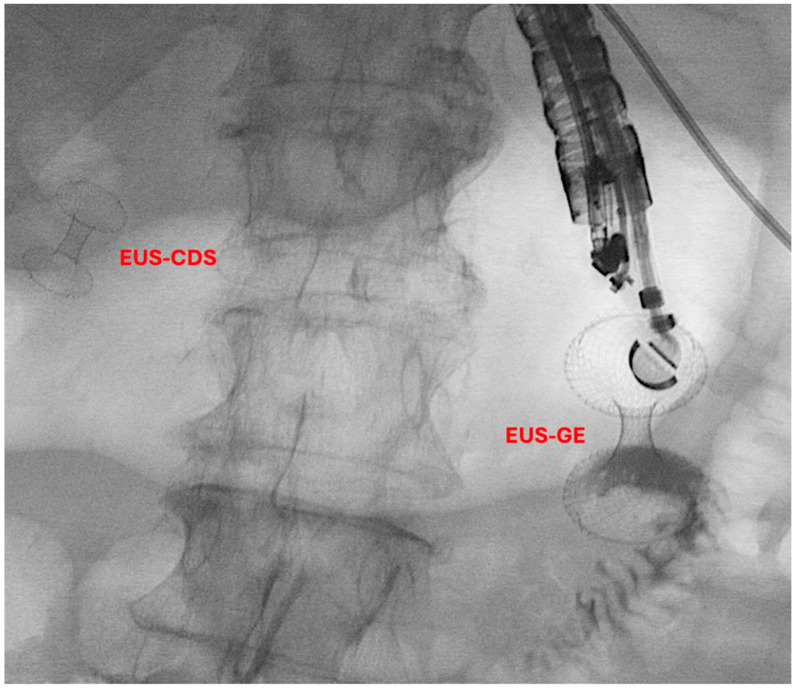
Fluoroscopic image of a patient presenting with malignant double obstruction (biliary and gastric outlet) wherein Endoscopic Ultrasound-Guided Choledochoduodenostomy (EUS-CDS) and Endoscopic Ultrasound-Guided Gastroenterostomy (EUS-GE) have been performed (permission has been taken from Dr. Jayanta Samanta for the publication of this image). The copyright of the image belongs to the authors.

If the duodenal bulb is infiltrated by neoplastic tissue with wall involvement, EUS-CDS and EUS-GBD via the duodenal route may not be performed safely (Figure 6).

**Figure 7:**
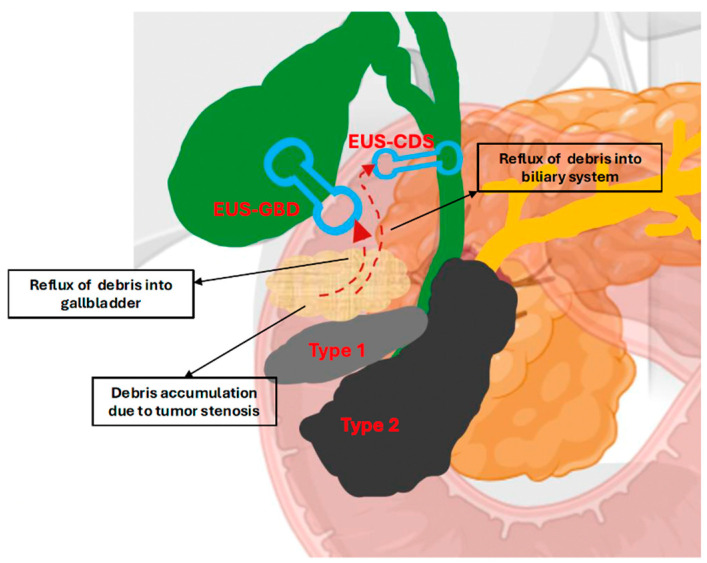
Schematic representation of ascending cholangitis/cholecystitis risk in patients with distal malignant biliary obstruction combined with type I or type II duodenal stenosis (according to Mutignani M et al. Classification [83]), treated by endoscopic ultrasound-guided gallbladder drainage (EUS-GBD) and Endoscopic Ultrasound-Guided Choledochoduodenostomy (EUS-CDS). The copyright of the image belongs to the authors.

If the stenosis is confined to the upper duodenal genu, the primary limitation of performing EUS-CDS and EUS-GBD through the duodenal route is the risk of ascending cholangitis, mainly due to the reflux of food and secretions from the duodenal bulb into the CBD or gallbladder (Figure 7) [23,24].

In patients with gallbladder in situ, another option could be performing EUS-GBD via the gastric route. Typically, in cases of dMBO, the gallbladder is distended, making it feasible to identify an EUS window through the gastric antrum suitable for the free-hand release of LAMS for EUS-GBD. It is crucial to thoroughly evaluate with EUS, particularly for potential cystic duct involvement by the tumor, and to assess the distance between the neoplastic lesion and the cystic duct. If the cancer is very close to the cystic duct, EUS-GBD may not provide long-term relief from jaundice [47,51].

**Figure 8:**
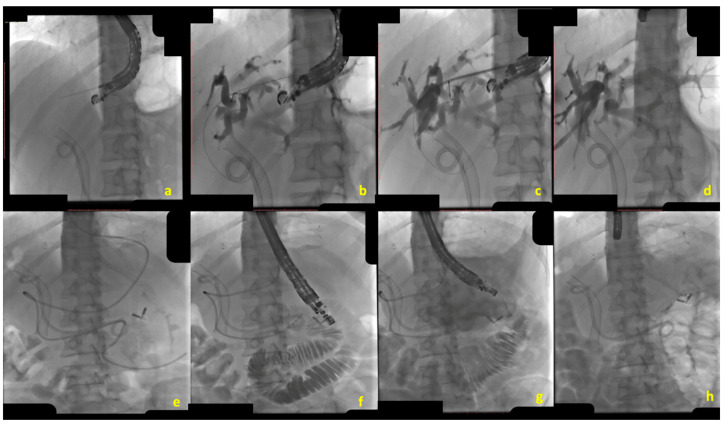
Figure illustration of the procedure Endoscopic Ultrasound-Guided Hepaticogastrostomy (EUS-HGS) with Endoscopic Ultrasound-Guided Gastroenterostomy (EUS-GE). A patient was diagnosed with metastatic gallbladder carcinoma and presented with jaundice. (**a**) Fluoroscopic image showing a metal self-expandable metal stent (SEMS) with coaxial double pigtail plastic stent in situ. The intrahepatic biliary radicle (left ductal system) was punctured with a 19-G Endoscopic Ultrasound Fine Needle Biopsy (EUS-FNB) needle; (**b**) following duct puncture, a cholangiogram was performed, and a guidewire was passed and coiled in the ductal system; (**c**) after adequate positioning of the guidewire, the tract was dilated with 6 French cystotomes; (**d**) placement of a partially covered SEMS (PC-SEMS), completing EUS-HGS procedure; (**e**) patient presented with symptoms of gastric outlet obstruction after 3 months; he was planned for EUS-GE; fluoroscopic image shows EUS-HGS PC-SEMS and biliary SEMS (with coaxial plastic stent) in situ. A nasojejunal (NJ) tube has been passed across the duodenal obstruction with the distal end in the first jejunal loop; (**f**) linear echoendoscope introduced and jejunal loops filled with contrast through NJ tube for delineation; (**g**) EUS-GE performed using 20 × 10 mm Lumen-Apposing Metal Stent (LAMS) using the free-hand technique; and (**h**) final fluoroscopic images showing all the stents (EUS-HGS stent, EUS-GE stent, and prior placed biliary SEMS with coaxial plastic stent) (permission has been taken from Dr. Jayanta Samanta for the publication of this image). The copyright of the image belongs to the authors.

Furthermore, EUS-GBD via the gastric route is limited by two potential complications: food impaction and LAMS-buried syndrome. For the former, placing a prophylactic double pigtail plastic stent (DPPS) may help prevent food occlusion, although there is currently no strong data supporting this approach [50]. The latter complication, LAMS-buried syndrome, arises from the properties and thickness of the gastric wall [84,85]. In this context, intrahepatic EUS-BD procedures, particularly EUS-HGS and EUS-AS, could provide two valuable and long-lasting solutions (Figure 8) [37,42].

In the bridge-to-surgery scenario, EUS-AS can offer biliary drainage without altering the anatomical route. By using EUS to access the left intrahepatic bile ducts, a biliary SEMS can be advanced in an antegrade transpapillary manner, achieving the same results as ERCP. For patients deemed unresectable and candidates for chemotherapy alone, EUS-HGS, alongside EUS-AS, offers another valuable solution. EUS-HGS, by placing a dedicated PC-SEMS, allows biliary drainage by connecting the left intrahepatic bile ducts to the stomach. Compared to EUS-AS, EUS-HGS has two key advantages: the ability to revise the PC-SEMS in case of obstruction with recurrence of jaundice or cholangitis and the potential to perform bridge stenting to drain the right bile ducts in cases of hilar stenosis with separation of the two biliary systems. Recently, the combination of EUS-HGS and EUS-AS has been proposed to provide a dual route for biliary drainage, aiming to reduce the risk of recurrence [39]. The two main limitations of intrahepatic EUS-BD procedures (EUS-HGS and EUS-AS) are primarily technical. First, adequate dilation of the left intrahepatic bile ducts is essential, along with the absence of massive ascites and neoplastic infiltration of the gastric wall. Secondly, both procedures are technically challenging and require highly experienced operators, including endoscopists and nursing staff, with proficiency in EUS and ERCP [9,33].

### 6.2. Distal Malignant Biliary Obstruction and Type II Gastric Outlet Obstruction

Type II GOO is likely the most challenging scenario due to the involvement of the papilla by neoplastic stenosis and infiltration. In this setting, endoscopists may encounter two main scenarios: naïve patients with DO or patients who have previously undergone ERCP with biliary SEMS placement and subsequently developed GOO and jaundice recurrence/cholangitis due to neoplastic progression and SEMS dysfunction, respectively [8]. In naïve patients, the preferred treatment for GOO in tertiary referral centers should be EUS-GE, with ES reserved for cases with technical contraindications or secondary centers [13]. Even when duodenal stenosis is passable with the duodenoscope, performing ERCP can be extremely challenging if the ampullary region is completely infiltrated by neoplastic tissue. Therefore, it may be necessary to consider an alternative route for biliary drainage. The comorbidities associated with PTBD should be reserved for select cases, making EUS-BD the standard of care in this scenario [86]. As mentioned, recent evidence has shown that EUS-CDS in this context is limited by the risk of ascending cholangitis, making it more suitable as a second-line strategy [23,24,51].

In contrast, EUS-BD through intrahepatic access, such as EUS-HGS and EUS-AS, appears to provide more durable jaundice relief with a comparable safety profile and could be considered a first-line approach for unresectable and resectable patients, respectively [24,43]. In patients with cholangitis who have previously placed biliary SEMS and subsequently developed duodenal type II stenosis (with or without ES in place), EUS-IBD (EUS-HGS, EUS-AS) could provide a valid alternative, as it not only enables biliary drainage but also allows for SEMS revision in an antegrade fashion [37]. Double stenting (ES and through-the-mesh biliary SEMS via ERCP) may serve as a rescue treatment strategy when therapeutic EUS procedures are unavailable. However, this “historical” approach has several limitations: the demonstrated superiority of EUS-GE over ES in terms of long-term efficacy, the need for two separate procedures (ERCP with SEMS placement following ES placement and expansion), and the technical challenges associated with cannulation and SEMS reinterventions.

### 6.3. Distal Malignant Biliary Obstruction and Type III Gastric Outlet Obstruction

In type III duodenal stenosis, the obstruction involves the third part of the duodenum without involvement of the papilla. In this case, transpapillary stenting by ERCP or EUS-AS is feasible, but distal duodenal stenosis could favor duodenal–biliary reflux, especially in the case of EUS-CDS because the duodenal bulb could act as a reservoir for debris and secretions, as in type II duodenal stenosis setting [23,24]. From this perspective, EUS-HGS could be a valid alternative for performing intragastric biliary drainage, minimizing the risk of duodenal–biliary reflux. Alternatively, if the duodenal stenosis is sufficiently distant from the papillary region or duodenal bulb, transpapillary stenting or EUS-CDS could be considered, especially if combined with an adjunctive ES, which could potentially reduce duodenal–biliary reflux.

## 7. Future Perspectives and Conclusions

Since its introduction in 2001, T-EUS has transformed the management of MDO, offering minimally invasive alternatives with significant clinical benefits, particularly in cases where ERCP fails or in patients with SAA. However, the standardization of T-EUS techniques, alongside structured training programs for endoscopists, remains crucial to achieving widespread implementation [9]. Notably, T-EUS procedures carry risks of severe complications, such as misdeployment and perforations, which endoscopists have not traditionally been trained to manage [67]. This underscores the importance of specialized training and the availability of multidisciplinary hospital resources, including interventional radiology and hepatopancreatobiliary (HPB) surgery. Further research on both short- and long-term complications is essential to establish standardized management strategies and enhance safety outcomes. Additionally, continued advancements in device technology could simplify procedures and further reduce the risk of AEs. A comprehensive evaluation of the financial implications of T-EUS, including long-term cost-efficacy analysis, is urgently needed to assess its economic viability and establish it as a cost-effective, front-line treatment for MDO. Such evaluations should not only consider the initial procedural costs but also the potential for reductions in hospital stays, reinterventions, and complication rates compared to surgical or percutaneous approaches. These analyses will provide critical insights into the broader economic impact of adopting T-EUS in routine clinical practice. Moreover, robust data are required to assess the compatibility of EUS-guided procedures with subsequent surgical interventions, such as curative resection or palliative bypass.

In conclusion, T-EUS represents a paradigm shift in the management of complex GOO and MBO, delivering effective palliation and expanding therapeutic options for patients with MDO. By balancing clinical efficacy with cost-effectiveness, fostering innovation in device technology, and promoting standardized techniques, T-EUS is well positioned to become the standard of care in the treatment of MDO.

## Figures and Tables

**Table 1:** Available evidence on endoscopic management of double obstruction (biliary and gastric outlet), including therapeutic endoscopic ultrasound procedures. ERCP, Endoscopic Retrograde Cholangiopancreatography. EUS-BD, EUS-guided biliary drainage. EUS-GE, EUS-guided gastroenterostomy. EUS-CDS, EUS-Guided Choledochoduodenostomy. EUS-HGS, EUS-Guided Hepaticogastrostomy. EUS-AS, EUS-Guided Antegrade Stenting. EUS-GBD, EUS-guided gallbladder drainage. ES, enteral stent. GOO, gastric outlet obstruction. EUS-RVS, Endoscopic Ultrasound-Assisted Rendez-Vous. TPS, transpapillary stenting. S-GE, surgical gastroenterostomy. S-HJ, Surgical Hepaticojejunostomy.

Study	Centers/ Design	No. of Patients	Treatment of GOO	Type of BD	Time Between Procedures	Technical Success (%)	Clinical Success (%)	Adverse Events (%)	Mean Stent Patency (d, Days)	Stent Dysfunction/ Recurrence (%)
Ogura, T., et al. (2016) [76]	Single-center, Retrospective	39	ES	EUS-CDS 33%; EUS-HGS 67%	Within 7 days	ES 100% EUS-BD 100%	ES 100% EUS-BD 100%	20.5% (15.5% EUS-CDS; 5% EUS-HGS)	ES: EUS-CDS/EUS-HGS 42 d/113 d; *p* = 0.343 BD: EUS-CDS/EUS-HGS 43 d/133 d; *p* = 0.0497	NA
Hamada, T., et al. (2018) [77]	Multicenter, Retrospective	110	ES	ERCP 82%; EUS-CDS 9.1%; EUS-HGS 9.1%	Simultaneous or within 7 days	ES 100% BD 95%	NA	14% (ERCP 8.9%, EUS-CDS 20% EUS-HGS 50%; *p* = 0.0029)	ERCP/EUS-CDS/EUS-HGS 455 d/344 d/137	34%
Debourdeau, A., et al. (2021) [78]	Single-center, Retrospective	31	ES	ERCP 25.8%; EUS-HGS 35.5%; EUS-CDS 3.2%; PTBD 35.5%	Simultaneous or within 7 days	ES NA BD 100%	ESNA BD 100%	42%	NA	NA
Mangiavillano, B., et al. (2021) [79]	Multicenter, Retrospective	23	ES	EUS-GBD 60.9%; EUS- CDS 39.1%	Simultaneous or within 7 days	ES 100% EUS-BD 95.6%	ES 100% EUS-BD 100%	0%	NA	NA
Canakis, A., et al. (2022) [80]	Multicenter, Retrospective	23	EUS-GE	EUS-HGS	Simultaneous	EUS-GE 100% EUS-HGS 95.6%	EUS-GE 100% EUS-HGS 72.7%	21.7%	NA	14%
Vanella, G., et al. (2023) [23]	Multicenter, Retrospective	93	ES 52.7% EUS-GE 42.3%	TPS 61.3% EUS-HGS 7.5% EUS-CDS 31.2%	Simultaneous or within 68 days	ES+TPS/ ES+EUS-CDS/ EUS-GE+EUS-HGS/ EUS-GE+TPS 100% ES+EUS-HGS 0% EUS-GE+EUS-CD 95%	ES+TPS 84.2% ES+EUS-CDS 60% EUS-GE+TPS 96.6% EUS-GE+EUS-CD 88.9% EUS-GE+EUS-HGS 83.3%	ES+TPS 18.4% ES+EUS-CDS 20% ES+EUS-HGS 100% EUS-GE+TPS 24.1% EUS-GE+EUS-CD 26.3% EUS-GE+EUS-HGS 16.7%	NA	EUS-CDS 28.1%. EUS-HGS 0%. TPS 71.9%. ES 68.7%; EUS-GE 31.2%
Bronswijk, M., et al. (2023) [81]	Multicenter, Retrospective	154	EUS-GE 34% S-GE 66%	EUS-BD 34% (EUS-CDS, EUS-HGS EUS-AS, EUS-RVS) S-HJ 66%	Simultaneous	EUS 96.2% SURGERY 100%	EUS 94.1% SURGERY 82.2% *p* = 0.049	EUS 11.3% SURGERY 34.7% *p* = 0.002	NA	NA
Fugazza, A., et al. (2024) [82]	Multicenter, Retrospective	77	ES 67.5% EUS-GE 32.5%	EUS-CDS	GOO treatment after a mean time of 14.3 ± 4.5 days	ES 100% EUS-GE 96% EUS-CDS 100%	ES 98% EUS-GE 100% EUS-CDS NA	14.5% (EUS-GE 8%) ES 17.3%, *p* = 0.27)	NA	Biliary 14.5% GOO 11.5%
Vanella, G., et al. (2024) [24]	Single-center, Prospective	20	EUS-GE	EUS-CDS 35%; EUS-HGS 65%	Simultaneous or within 38 days	EUS-GE 95% EUS-BD 100%	EUS-GE 95% EUS-BD 100%	20% EUS-CDS 42.9%; EUS-HGS 7.7%, *p* = 0.067	NA	EUS-CDS 71.4%; EUS-HGS 16.7%, *p* = 0.02
